# Deciphering the guest-free crystal structures and thermal breathing of the flexible metal–organic frameworks ZIF-7 and ZIF-9

**DOI:** 10.1039/d5sc08614k

**Published:** 2026-02-09

**Authors:** Athanasios Koutsianos, Erik Svensson Grape, Roman Pallach, Julian Keupp, Rochus Schmid, A. Ken Inge, Sebastian Henke

**Affiliations:** a Anorganische Chemie, Fakultät für Chemie und Chemische Biologie, Technische Universität Dortmund Otto-Hahn-Straße 6 44227 Dortmund Germany sebastian.henke@tu-dortmund.de; b Wallenberg Initiative Materials Science for Sustainability, Department of Chemistry, Stockholm University 10691 Stockholm Sweden; c Fakultät für Chemie und Biochemie, Ruhr-Universität Bochum Universitätsstraße 150 44801 Bochum Germany

## Abstract

Owing to their dynamic phase behaviour and unique gas sorption properties, flexible metal–organic frameworks (MOFs) have emerged as a promising class of materials for applications in gas-related technologies and beyond. Resolving the crystal structures of the distinct phases is essential for understanding their transformation mechanisms and rational tuning framework responsiveness. Here, we revisit the prototypical flexible MOFs ZIF-7 (Zn(bim)_2_, bim^−^ = benzimidazolate) and ZIF-9 (Co(bim)_2_) and resolve the long-standing ambiguity surrounding their guest-free narrow-pore (*np*) phases. Using microcrystal three-dimensional electron diffraction combined with powder X-ray diffraction (PXRD) and density functional theory calculations, we determine the crystal structures of both np phases. The results rectify previous structural models and incomplete structural descriptions of the *np* phase of ZIF-7 and establish the structure of the *np* phase of ZIF-9. In contrast to the high-symmetry, guest-accommodating large-pore (*lp*) phases, the np phases adopt distorted, densely packed frameworks with strongly deformed sodalite cages, reduced void fractions, and enhanced framework densities. Variable-temperature PXRD and differential scanning calorimetry further reveal metal-dependent anisotropic thermal expansion of the *np* phases and entropy-driven *np*–*lp* transitions.

## Abstract 2

We decipher the crystal structures of guest-free ZIF-7 and ZIF-9 by 3D electron diffraction, resolving prior ambiguity and establishing the narrow pore phases. Also, variable temperature XRD and calorimetry reveal entropy-driven thermal breathing.

## Graphical abstract

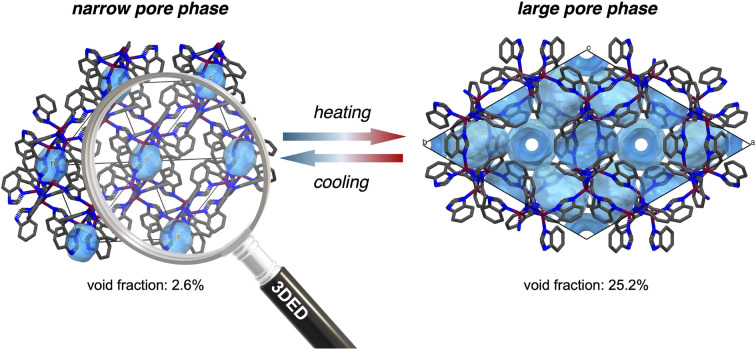

## Introduction

Zeolitic imidazolate frameworks (ZIFs) constitute an important subfamily of metal–organic frameworks (MOFs) that mimic network topologies of zeolites due to their structural resemblance.^1–3^ Their modular architecture, combined with exceptional thermal and chemical stability as well as facile synthesis, has driven intense research interest.^1,4^ Beyond their robustness, several ZIFs display framework flexibility,^5–8^ undergoing reversible structural transformations in response to external stimuli such as gas pressure, mechanical compression, or temperature.^9,10^ Notably, some ZIFs undergo gas-sorption-induced transitions, including gate-opening or breathing behaviour, whereby the pore size dynamically adapts upon gas sorption.^6,11,12^ This flexibility originates from a subtle balance between enthalpic (*e.g.* guest-framework and intra-framework interactions) and entropic (*e.g.* vibrational and configurational degrees of freedom) contributions.^13–15^

**Fig. 1:**
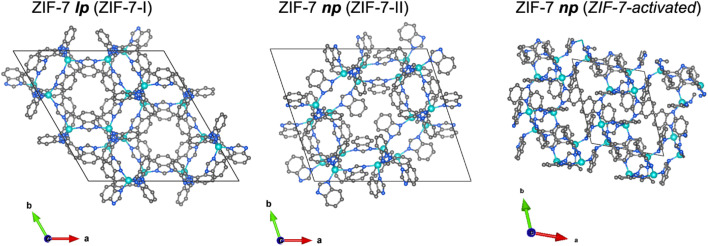
Illustrations of the crystal structures of the ZIF-7 *lp* phase (ZIF-7-I^1^) and the two ZIF-7 *np* phases (ZIF-7-II^29^ and ZIF-7 activated^21^) proposed in the literature. Zn, teal; N, blue; C, grey; hydrogen atoms are omitted for clarity.

ZIF-7 (Zn(bim)_2_, bim^−^ = benzimidazolate) is a canonical example of a flexible MOF, known for its reversible guest-induced phase transition between a narrow-pore (*np*) and a large-pore (*lp*) phase.^16^ The as-synthesized form adopts the *lp* phase, stabilized by occluded solvent molecules. It features a rhombohedrally distorted sodalite (sod) topology, constructed from tetrahedrally coordinated Zn^2+^ centers interconnected by bim^−^ linkers (space group: *R*3̄, Fig. 1 and Table 1).^1^ Upon desolvation, the framework transforms to a guest-free *np* phase. Besides guest removal, exposure to gases such as CO_2_, CH_4_, H_2_, and short-chain (C_2_–C_3_) hydrocarbons induces reopening of the structure, forming the *lp* phase at well-defined threshold pressures, as evidenced by sigmoidal gas sorption isotherms^17,18^ and *in situ* powder X-ray diffraction (PXRD) studies.^19^ In addition to guest-induced transformations, it has been shown that guest-free ZIF-7 also undergoes an *np*–*lp* transition upon heating.^19^ This thermally induced phase transition is driven by entropic stabilisation of the expanded *lp* phase, reflecting an adsorption-independent breathing mechanism. This responsive behaviour underpins ZIF-7's relevance for gas separation, storage and sensing applications.^17,20,21^ Specifically, the step-shaped isotherms associated with the *np*–*lp* transition render ZIF-7 particularly attractive for selective gas separation and pressure-swing adsorption processes.^22,23^

**Table 1:** Crystallographic data of the ZIF-7 *lp* phase (ZIF-7-I ^1^) and the two ZIF-7 *np* phases (ZIF-7-II ^29^ and ZIF-7 activated^21^) proposed in the literature. The void fractions have been calculated *via* Mercury software (probe radius 1.2 Å and grid spacing 0.2 Å)

Material	ZIF-7-I	ZIF-7-II	ZIF-7-activated
Phase model	*lp* phase	*np* phase	*np* phase
Space group	*R*3̄	*P*1̄	*P*1̄
*a*/Å	22.989(3)	23.948(6)	11.320(3)
*b*/Å	22.989(3)	21.354(6)	14.158(4)
*c*/Å	15.763(3)	16.349(4)	14.212(5)
*α*/°	90	90.28(2)	109.82(2)
*β*/°	90	93.275(17)	95.30(2)
*γ*/°	120	108.412(14)	109.459(19)
*V*/Å^3^	7214(2)	7917(3)	1965.5(11)
*Z*	18	2	2
Volume per Zn(bim)_2_ formula unit/Å^3^	400.77(11)	439.85(17)	327.6(2)
Void fraction/%	26.1	34.4	1.2
Density/g cm^−3^	1.24	1.13	1.51

Already more than 19 years ago, the crystal structure of the *lp* phase of ZIF-7 was determined by single-crystal X-ray diffraction (SCXRD), as large single crystals form under solvothermal synthesis conditions.^1^ In contrast, the guest-free *np* phase remains difficult to characterise structurally. Upon desolvation and formation of the *np* phase, the crystals fracture into small microparticles. Moreover, PXRD patterns of the *np* phase are characterised by substantial peak broadening, likely caused by size and strain effects, in combination with extensive Bragg reflection overlap due to low symmetry. These factors render structure determination from PXRD data highly challenging.

To realise gas separation and sensing applications under varying operational conditions, precise control of switching pressures is required.^24–26^ Metal substitution has emerged as an effective strategy to tune transition thermodynamics. In this context, isostructural analogues such as ZIF-9 (Co(bim)_2_) and CdIF-13 (Cd(bim)_2_) have been explored in comparison to ZIF-7 with regard to gas uptake and switching behaviour.^27,28^ Like ZIF-7, both materials exhibit reversible transitions between *np* and *lp* phases. Substituting Zn^2+^ with Co^2+^ or Cd^2+^ raises the threshold pressure for the gas-induced transition, emphasising the role of the metal node in modulating framework flexibility. Due to the similarity in PXRD patterns and gas uptake behaviour, structurally analogous *np* phases have been hypothesised for ZIF-7 and ZIF-9. However, the structure of guest-free ZIF-9 has remained unresolved.

Initial attempts to solve the *np* structure of the ZIF-7 relied on structural modeling and Rietveld refinement of PXRD data.^29^ The resulting model, ZIF-7-II (space group *P*1̄), is inconsistent with key experimental and computational observations. Notably, it exhibits a lower crystallographic density and a higher pore volume than the *lp* phase (Fig. 1 and Table 1) – an implausible scenario for an enthalpically stabilized contracted phase. The fact that molecular dynamics simulations predicted a higher crystal density for the *np* phase,^19^ alongside the inability of independent groups to reproduce the ZIF-7-II model by Rietveld refinement of PXRD data,^19,28^ raises further doubts. Despite these inconsistencies, the ZIF-7-II model has nonetheless been employed in experimental and computational studies to rationalise ZIF-7's sorption behaviour.^29,30^

In 2021 a study addressed this issue by fitting the powder neutron diffraction pattern of the *np* phase of ZIF-7 using a structural model derived from single-crystal X-ray diffraction (SCXRD) of the guest-free isoreticular analogue CdIF-13.^21,27^ The resulting structure, referred to as ZIF-7-activated (Fig. 1 and Table 1), represents a dense, chemically plausible triclinic phase (space group *P*1̄), consistent with prior simulation data and thermodynamic expectations. Importantly, unlike the ZIF-7-II model, this structure is denser than the *lp* phase, aligning with the expected features of a guest-free contracted framework. However, the reliability of this structural model is limited by the inherent challenges of neutron powder diffraction for low-symmetry, poorly crystalline phases. Severe Bragg peak broadening and overlap precluded a detailed refinement, necessitating the use of rigid-body constraints. Moreover, key structural features, such as atomic anisotropic displacement parameters (ADPs), could not be determined.

To overcome these limitations, we employ three-dimensional electron diffraction (3DED), an emerging technique,^31–41^ to resolve the crystal structures of the guest-free *np* phases of ZIF-7 and ZIF-9 from single crystal microparticles. 3DED enables high-quality reciprocal space data collection from submicron-sized single crystals, allowing detailed atomistic models to be derived. Comparative analysis reveals pronounced structural resemblance between ZIF-7 and ZIF-9, which is also reflected in their analogous thermal expansion and thermo-responsive breathing behaviour as established by variable-temperature (VT-)PXRD and differential scanning calorimetry (DSC). Density functional theory (DFT) calculations further support the derived structural models and elucidate the enthalpic stabilization of the *np* phases by dispersion interactions.

## Results and discussion

### Structural characterization

Following reported procedures,^28,42^ highly crystalline solvated crystals of ZIF-7 and ZIF-9 (*lp* phases) were synthesized, free of detectable impurities, as confirmed by PXRD (Fig. S1). Upon activation at 200 °C under dynamic vacuum, the materials converted into their guest-free *np* phases. These activated phases exhibited diminished crystallinity, broadened Bragg reflections, and extensive peak overlap, features consistent with previously reported PXRD patterns of the *np* phases (Fig. S1).

**Fig. 2:**
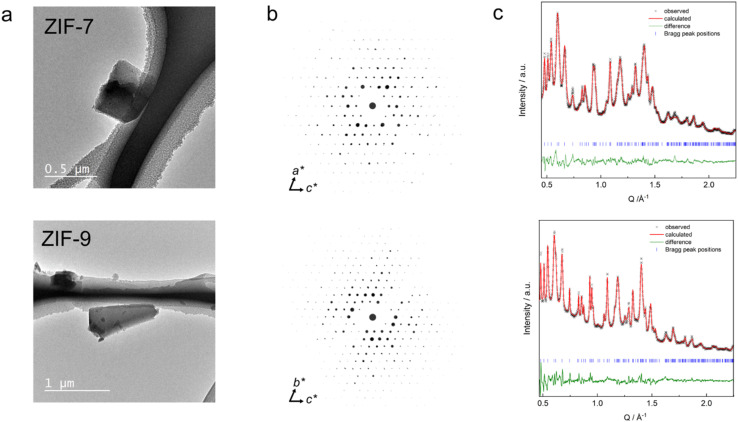
(a) TEM images of ZIF-7 (top)/ZIF-9 (bottom) crystals used for the collection of 3DED data. (b) Reconstructed full reciprocal space projections from 3DED data as viewed along b* (top) and a* (bottom), showing reflections as spheres of relative sizes corresponding to the observed intensity maxima. (c) Rietveld fits of ZIF-7/ZIF-9 *np* phases to PXRD data.

3DED data were collected from four individual submicron-sized single crystals of both ZIF-7 and ZIF-9, with rotational coverage of each dataset ranging from 75.2° to 94.7° for ZIF-7 and 82.2° to 94.5° for ZIF-9 (Fig. 2a, b and Table S2, see SI for experimental details). Indexing of the datasets provided triclinic unit cells (space group *P*1̄) with very similar unit cell parameters for both materials (Table S2), and data completeness values of 88% for ZIF-7 and 82% for ZIF-9. These results confirm the close structural analogy between the two frameworks. Moreover, the unit cell parameters and the atomic coordinates align well with the ZIF-7-activated structure previously determined from neutron diffraction data (Table 1), thereby validating that model and clearly distinguishing it from the earlier ZIF-7-II model.

**Fig. 3:**
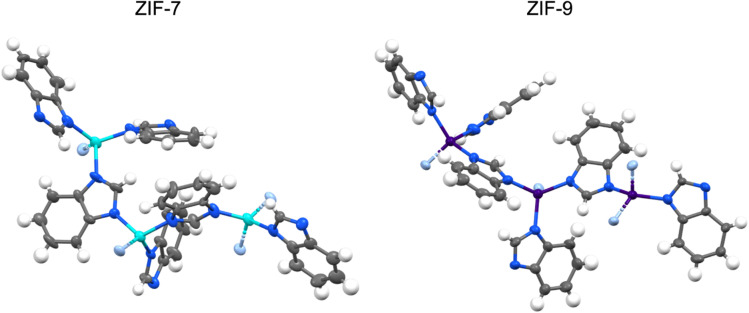
Ellipsoidal (50% probability) plots of the extended asymmetric units (metal atoms are shown with complete coordination environments) of ZIF-7 *np* (left) and ZIF-9 *np* (right) derived from 3DED. Zn, Co, C, N and H atoms are shown in teal, purple, grey, blue and white, respectively. Nitrogen atoms not belonging in the asymmetric unit are displayed in light blue.

The data quality of the 3DED data was sufficient to locate all hydrogen atoms from the Fourier difference maps^35^ and to refine all non-hydrogen atoms with atomic ADPs that reached physically sound components; a level of structural detail not achievable in the previous neutron diffraction study (Fig. 3). Notably, while the *lp* phases of both ZIFs feature only one crystallographically unique M^2+^ ion and two distinct bim^−^ linkers per asymmetric unit, the *np* phases exhibit three unique M^2+^ ions and six distinct bim^−^ linkers, reflecting their lower symmetry and higher structural complexity.

**Fig. 4:**
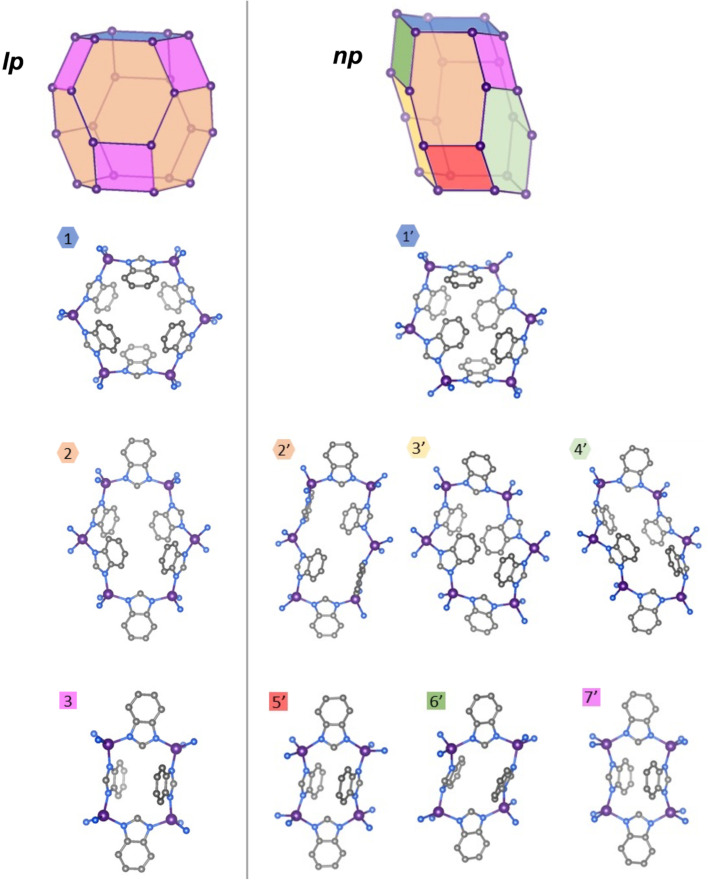
Structural representations of ZIF-9 *np* (right) sodalite-like cage and the unique four-membered rings and six-membered rings in comparison to the *lp* phase^1^ (left, CCDC VEJZEQ). Co, purple; N, blue; C, grey; hydrogen atoms are omitted for clarity.

Compared to the high-symmetry solvated *lp* phases, the *np* phases adopt collapsed, highly distorted conformations, resulting in a dense crystal packing and loss of void volume. The internal connectivity of the building units in the *np* phase is still characteristic of the sod topology, indicating that the phase transition maintains chemical bonding within the framework. To visualise these structural differences between the *lp* and *np* phases, we constructed a graphical representation based on a single sodalite-type cage (a distorted truncated octahedron), which serves as the repeating unit of the network structure (the network is based on a body-centred packing of these sodalite-type cages). Since the structural differences between both phases of ZIF-7 and ZIF-9 are minimal, only ZIF-9 structures are presented here for clarity; the corresponding ZIF-7 structures are provided in the SI (Fig. S5). The vertex representations of the sodalite cages, as well as atomistic representations of the individual ring configurations of the *lp* and *np* phases, are set side by side to better visualise the structural differences between the two phases (Fig. 4). In the *lp* phase, the sodalite network exhibits a rhombohedral distortion (space group *R*3̄) from the ideal cubic *Im*3̄*m* symmetry due to the bulk of the bim^−^ linkers and their arrangement in the network. Conversely, the *lp* phase features two distinct types of 6-membered rings (1 and 2), occurring in a 1 : 3 ratio, and one type of 4-membered ring (3), all arranged around a 3̄ axis, generating the rhombohedrally distorted sodalite cage. Upon transition to the *np* phase (space group *P*1̄), the point symmetry of the sodalite cage is reduced to 1̄, resulting in four unique 6-membered rings (1′, 2′, 3′, 4′) and three distinct 4-membered rings (5′, 6′, 7′). In contrast, the previous study reporting the ZIF-7-activated phase erroneously proposed that the *np* structure of ZIF-7 contains only three unique 6-membered rings and a single 4-membered ring, overlooking three additional distinct rings that emerge upon symmetry reduction.^21^

Comparison of the ring conformations in the *lp* and *np* phases reveals pronounced distortions in the *np* structures, particularly among the 6-membered rings. These undergo significant asymmetric elongation and compression, accompanied by substantial rotations of some bim^−^ linkers about the M–bim–M connection axes, as well as out-of-plane twisting relative to the M–bim–M planes. These cooperative deformations result in a denser packing of the framework building units. Some of the 4-membered rings also exhibit marked deviations from their original geometries. For instance, ring 6′ adopts an oblique rhombic configuration in contrast to the nearly square-shaped ring 3 in the *lp* phase, reflecting the overall compression of the sodalite cage. Such structural distortions enhance van der Waals contacts between adjacent linkers, thereby contributing to the enthalpic stabilisation of the guest-free *np* phase.

**Fig. 5:**
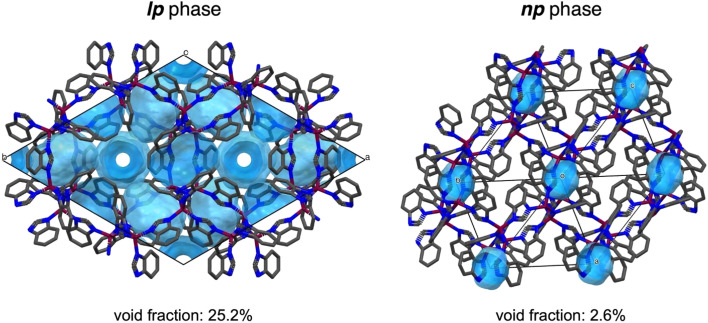
Representation of the void space in the *lp* phase (CCDC VEJZEQ)^1^ and *np* phase of ZIF-9 (CCDC deposition code: 2493471). The voids are calculated with a probe radius of 1.2 Å and a grid spacing of 0.2 Å. A similar viewing direction of the frameworks was attempted (along [001] for the *lp* phase and along [111] for the *np* phase). For the *lp* phase, atoms from solvent guests have been removed. For the *np* phase, the structure from the Rietveld refinement is taken with H atoms added at idealised positions for the void space calculation. Co, purple; N, blue; C, grey; hydrogen atoms are omitted for clarity. The void space is highlighted in light blue. Unit cell boundaries are shown as thin black lines.

The structural models derived from 3DED were verified by Rietveld refinement of the models against high-resolution PXRD data recorded at room temperature (Fig. 2c). Due to the low symmetry, severe peak overlap, and peak broadening, the Rietveld model used rigid bodies for the bim^−^ linkers and applied distance and angle restraints for the [MN_4_] tetrahedra. H atoms were omitted from the Rietveld fit, due to their minimal contribution to the PXRD patterns. Notably, the lattice parameters obtained from the Rietveld fits of the PXRD data (Table S3) are 0.5% to 4.3% smaller than those derived from 3DED (Table S2), resulting in significantly smaller unit cell volumes from PXRD data. Specifically, the unit cell volume of ZIF-7 from PXRD is 8.8% smaller, and that of ZIF-9 is 5.2% smaller compared to 3DED. This difference cannot be attributed to the measurement temperatures (98 K for 3DED *vs.* 25 °C for PXRD), as lower temperatures would be expected to yield smaller unit cell volumes (see discussion about the thermal expansion behaviour of ZIF-9 and ZIF-9 below). We therefore attribute the discrepancy to the lower accuracy and precision of the unit cell parameters obtained from 3DED due to various experimental challenges associated with the instrument.^43^ Hence, we use the crystallographic parameters from the PXRD data refinements for the subsequent discussion of the variations in framework volumes and pore volumes. The volumes per M(bim)_2_ formula unit extracted from PXRD data change from 406.12(3) Å and 406.05(2) Å for the *lp* phases of ZIF-7 and ZIF-9 to 339.9(5) Å and 332.99(5) Å for the *np* phases, reflecting a compression by 16% (ZIF-7) and 18% (ZIF-9), respectively. Using the void calculation routine of the software Mercury (probe radius 1.2 Å and grid spacing 0.2 Å), the *np* phases feature only 3.5% void fraction for ZIF-7 and 2.6% for ZIF-9 (Fig. 5). This is in stark contrast to the reported *lp* phases, which possess a void fraction of 26.1% for ZIF-7 and 25.2% for ZIF-9 (neglecting solvent guests in the calculation). Remarkably, the obtained crystallographic densities equal 1.41 g cm^−3^ for ZIF-7 and 1.42 g cm^−3^ for ZIF-9, essentially identical to the density estimated from molecular dynamics simulations,^19^ reporting a value of 1.42 g cm^−3^.

The isolated, tiny pores of the *np* phases are further in agreement with the well-known CO_2_ adsorption behaviour of ZIF-7 and ZIF-9, which exhibit extremely low gas uptake prior to the *np*–*lp* phase transformation.^28^ Moreover, the structural validity of the *np* phases is verified by dispersion corrected periodic DFT calculations using the QUICKSTEP/CP2K code^44^ at a level of theory established for such systems^45^ (for details see SI). Zero Kelvin optimizations of guest-free ZIF-7, starting from structural models of the *lp* and the *np* phase, resulted in local minima, with the compact *np* phase energetically favoured by about 12 kJ mol^−1^ per Zn(bim)_2_ formula unit. Moreover, a geometry optimisation starting from the previously reported ZIF-7-II phase does not stabilize to a nearby local minimum, but instead converges to the geometry found for the *lp* phase, highlighting that the proposed ZIF-7-II phase is not a local minimum at this level of theory.

To rationalise the energetic preference for the *np* phase, we performed a Hirshfeld surface analysis based on the DFT-optimised *lp* and *np* structures of ZIF-7 (Fig. S6). The *d*_norm_-mapped Hirshfeld surfaces (where *d*_norm_ is the normalised contact distance relative to the sum of van der Waals radii, highlighting contacts close to the van der Waals separation) and the corresponding fingerprint plots (Fig. S7 and Table S4) show a clear shift towards shorter inter-linker (non-bonded) contacts in the *np* phase, consistent with a markedly tighter packing of the framework. In particular, the *np* structure exhibits substantially shorter H⋯H and C⋯H contacts between neighbouring linkers, indicative of enhanced dispersion-dominated stabilisation and more pronounced C–H⋯π interactions compared to the *lp* phase.

### Thermal phase behaviour

Previous work has shown that guest-free ZIF-7 can undergo a thermally-induced, entropy-driven transition from the *np* to the *lp* phase between 500 and 700 °C, depending on the atmosphere (CO_2_, N_2_, or vacuum).^19^ The high-temperature *lp* phase closely resembles the low-temperature, guest-occupied *lp* phase, but has a higher enthalpy than the *np* phase. Motivated by the close structural analogy between ZIF-7 and ZIF-9, we investigated whether ZIF-9 exhibits a similar thermoresponsive behaviour and compared its response to that of ZIF-7.

**Fig. 6:**
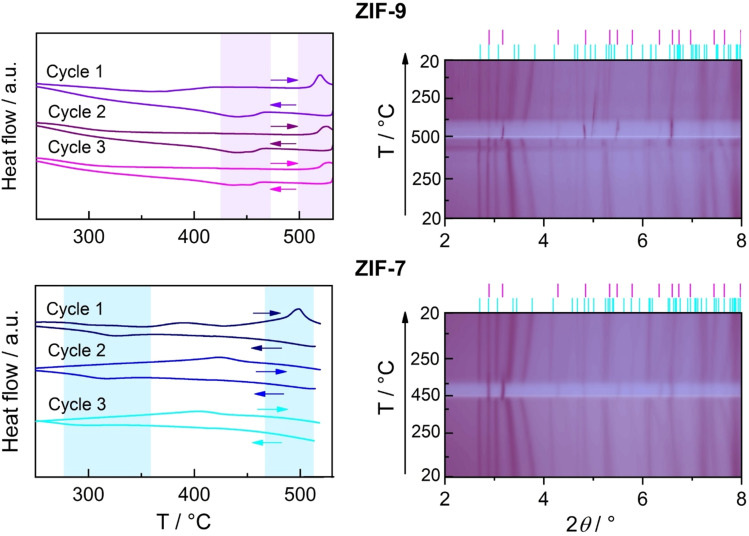
Thermo-responsive behaviour of ZIF-9 (top) and ZIF-7 (bottom) materials. Left: DSC traces of the ZIFs under N_2_ atmosphere for three consecutive heating/cooling cycles with a heating/cooling rate of 10 °C min^−1^. Regions in light blue (ZIF-7) and violet (ZIF-9) highlight the observed peaks. Right: Contour maps of the VT-PXRD patterns. The colour grading from light to dark indicates increasing intensity. In the case of ZIF-9, the map comprises the heating section, from 25 to 495 °C, generated from 13 patterns and the cooling section, from 495 to 25 °C, generated from 13 patterns. The full dataset is provided in Fig. S17 and S18. For ZIF-7 the map comprises the heating section, from 25 to 451 °C, generated from 17 patterns, and the cooling section, from 451 to 25 °C, generated from 13 patterns. The full dataset is provided in Fig. S19 and S20.

For ZIF-9, DSC measurements on activated samples over three heating/cooling cycles revealed a distinct endothermic peak in the first upscan, with an onset at ∼512 °C (Fig. 6). TGA traces show negligible mass loss up to this temperature, indicating that the signal arises from a thermally-driven *np*–*lp* transition. Above ∼535 °C, pronounced mass loss indicates the onset of decomposition,^42^ prompting us to set 535 °C as the upper temperature limit for cycling experiments. The *np*–*lp* transition enthalpy (Δ*H_np_*_–_*_lp_*) from the first upscan is ∼1.6 kJ mol^−1^ (Table S5). Upon subsequent cooling from 535 °C back to 200 °C, the reverse exothermic *lp*–*np* transition is observed around 465 °C, giving a lower enthalpy (Δ*H_lp_*_–_*_np_* ∼ −1.3 kJ mol^−1^), likely due to signal broadening and/or partial decomposition of the material at high temperatures. The pronounced shift of the transition temperature for the reverse transition is characteristic of a first-order phase transition and also in line with the pressure hysteresis observed in gas sorption isotherms involving the related, guest-induced *np*–*lp* phase transition.^17,46^ In subsequent cycles, the onset temperature of the *np*–*lp* transition signal increases slightly, so that incomplete baseline recovery up to the maximum temperature of 535 °C prevented reliable determination of Δ*H_np_*_–_*_lp_* by peak integration. Cooling cycles 2 and 3 yielded Δ*H_lp_*_–_*_np_* values 17% and 33% lower in magnitude than in cycle 1, and displayed slightly reduced onset temperatures, consistent with progressive framework degradation during thermal cycling.

Variable temperature (VT)-PXRD measurements on ZIF-9 corroborate the DSC results. The *np*–*lp* phase transition is clearly visible around 480 °C (Fig. 6 and S17) and confirmed by profile fitting (Fig. S9 and Table S6). The transition temperature is notably reduced compared to the DSC experiment, which can be attributed to the much slower effective heating rate of the VT-PXRD experiment (4–5 min for X-ray exposure and detector readout per pattern) relative to DSC (10 °C min^−1^).^47^ Progressive shifts of the *np* phase reflections prior to the phase transition indicate strong thermal expansion of this phase. Pawley fitting^48^ of the VT-PXRD patterns (Fig. S9 and Table S6) and subsequent analysis of the derived temperature-dependent lattice parameters with the PAScal software package^49^ reveal that the framework contracts along the long axis of the stretched sodalite cages as the temperature increases (the coefficient of thermal expansion, CTE, is −22 MK^−1^), while expanding in the perpendicular directions (CTEs are +22 M K^−1^ and +138 M K^−1^; Tables S7 and S8, Fig. S10 and S11). The negative thermal expansion (NTE) behaviour of the *np* phase is characteristic of its structural evolution towards the rhombohedral *lp* phase, which features a less stretched sodalite cage (Fig. 4). Upon cooling, the reverse *lp*–*np* transition starts at ∼415 °C (Fig. S18) and is completed by ∼350 °C, again showing hysteresis relative to heating (Fig. 6).

Complementary DSC and VT-PXRD analyses of ZIF-7 confirm analogous behaviour, but at lower transition temperatures. DSC shows a rather broad endotherm with an onset at 471 °C (Δ*H_np_*_–_*_lp_* = 1.6 kJ mol^−1^), and VT-PXRD reveals the *np*–*lp* transition at ∼450 °C (Fig. 6, S12 and S19). The lower transition temperature of ZIF-7 compared to ZIF-9 aligns with the lower pressures required for the CO_2_-induced *np*–*lp* transition in ZIF-7, reflecting the higher relative stability of the *np* phase in ZIF-9.^28,50^ Notably, a broad exotherm at ∼380 °C precedes the *np*–*lp* transition in DSC and coincides with a small weight-loss in TGA (Fig. S8), suggesting this feature may be ascribed to partial decomposition. The thermal expansion behaviour of the *np* phase of ZIF-7 prior to the phase transition is likewise anisotropic (Tables S10 and S11, Fig. S13 and S14), featuring NTE analogous to ZIF-9 (CTEs are −16 M K^−1^, +10 M K^−1^ and +178 M K^−1^). On cooling, the exothermic *lp*–*np* transition is observed with an onset at 384 °C in DSC and ∼380 °C in VT-PXRD (Fig. 6 and S20). The broadened exothermic *lp*–*np* peak in DSC precluded reliable enthalpy determination. This may either reflect sluggish phase transition kinetics in ZIF-7 or progressive decomposition during thermal cycling. The latter is consistent with diminished and shifted phase transition signatures in subsequent DSC cycles (Table S5); however, VT-PXRD shows no indication of ZIF-7 decomposition after one single heating–cooling cycle.

## Conclusions

Combining 3DED and PXRD with DFT calculations enabled the structural resolution of the elusive guest-free phases of ZIF-7 and ZIF-9. In the absence of guest molecules, both frameworks adopt dense, collapsed *np* structures that crystallise in the triclinic *P*1̄ space group. These phases exhibit pronounced structural distortions compared to the higher symmetry *lp* forms, with strongly deformed sodalite cages, minimised void fractions, and increased framework densities. Such distortions account for the sigmoidal gas sorption behaviour widely reported for these materials.

The comprehensive structural analysis corrects earlier misassignments of the *np* phase of ZIF-7 and demonstrates that the dense forms are stabilised by cooperative framework deformations and enhanced van der Waals contacts between linkers. Comparison of ZIF-7 and ZIF-9 reveals their close structural analogy, while VT-PXRD and DSC highlight strong similarities in their thermal expansion behaviour and subtle differences in the thermally induced, entropy-driven *np*–*lp* phase transitions, signifying variations in the relative stabilities of their *np* and *lp* phases.

Altogether, this study provides a coherent structural picture of the guest-free *np* phases of ZIF-7 and ZIF-9, resolves longstanding ambiguities in their description, and provides direct insight into their temperature-induced responsiveness. More broadly, it showcases the power of 3DED to unravel complex MOF structures that remain difficult to access by conventional X-ray diffraction techniques, and underscores the value of combining calorimetric methods with *in situ* diffraction studies to understand transformation mechanisms of flexible MOFs.

## Author contributions

AK: formal analysis, investigation, validation, visualization, writing – original draft, writing – review & editing. ESG: formal analysis, investigation, writing – review & editing. RP: formal analysis, investigation, writing – review & editing. JK: formal analysis, investigation. RS: validation, resources, supervision, writing – review & editing. AKI: validation, resources, supervision, writing – review & editing. SH: conceptualization, funding acquisition, project administration, validation, resources, supervision, writing – review & editing.

## Conflicts of interest

There are no conflicts to declare.

## Supplementary Material

SC-017-D5SC08614K-s001

SC-017-D5SC08614K-s002

## Data Availability

CCDC 2493469–2493472 contain the supplementary crystallographic data for this paper.^51*a*–*d*^ The data supporting this article have been included as part of the supplementary information (SI). Supplementary information: include experimental and methodogical details, synthesis protocols, additional PXRD, ^1^H NMR, DFT, TGA and DSC data, and Hirshfeld surface analyses. See DOI: https://doi.org/10.1039/d5sc08614k.
