# Supplementary material for: Deciphering the guest-free crystal structures and thermal breathing of the flexible metal–organic frameworks ZIF-7 and ZIF-9
Source: Chem Sci. 2026 Feb 9;17(14):7185–93. doi: 10.1039/d5sc08614k (PMC12911875; doi:10.1039/d5sc08614k)
Supplement: SC-017-D5SC08614K-s001 [file SC-017-D5SC08614K-s001.pdf]

Supplementary Information (SI) for Chemical Science.
This journal is © The Royal Society of Chemistry 2026

Supplementary Information

Deciphering the guest-free crystal structures and thermal breathing of the flexible metal-organic frameworks ZIF-7 and ZIF-9

Athanasios Koutsianos[1], Erik Svensson Grape[2,#], Roman Pallach[1], Julian Keupp[3], Rochus Schmid[3], A. Ken Inge[2], Sebastian Henke[1]*

[1] Anorganische Chemie, Fakultät für Chemie und Chemische Biologie, Technische Universität Dortmund, Otto-Hahn-Straße 6, 44227 Dortmund, Germany. Email: sebastian.henke@tu-dortmund.de

[2] Wallenberg Initiative Materials Science for Sustainability, Department of Chemistry, Stockholm University, 10691 Stockholm, Sweden

[3] Fakultät für Chemie und Biochemie, Ruhr-Universität Bochum, Universitätsstraße 150, 44801 Bochum, Germany

[#] Current affiliation: Department of Chemistry - Ångström Laboratory, Uppsala University, 75120 Uppsala, Sweden

Contents

S1 Methods 2
S1.1 Three-dimensional electron diffraction (3DED) 2
S1.2 Powder X-ray diffraction (PXRD) 2
S1.3 Rietveld refinement of PXRD data 3
S1.4 Density functional theory (DFT) calculations 3
S1.5 Thermogravimetric analysis (TGA) 4
S1.6 Differential scanning calorimetry (DSC) 4
S1.7 ^{1}H NMR spectroscopy 5
S2 Materials synthesis 6
S2.1 ZIF-7 6
S2.2 ZIF-9 6
S3 Materials characterization 7
S3.1 Laboratory PXRD data 7
S3.2 ^{1}H NMR spectra 8
S3.3 3DED and Rietveld refinement data 10
S3.4 Hirshfeld surface analysis of DFT-optimized structures 14
S3.5 TGA data 16
S3.6 DSC and VT-PXRD data 17
S4 References 34

S1 Methods

S1.1 Three-dimensional electron diffraction (3DED)

Samples of ZIF-7 and ZIF-9 powders were activated (see Sections S2.1 and S2.2), and subsequently added as dry powders (to retain the ***np*** state) in great excess (>> 1 mg) onto holey carbon grids. Any powder not adhering to the holey carbon film was then subsequently removed by blowing air onto the grids exposed to the powders. The as-prepared samples were then loaded in a Gatan 914 cryo-transfer holder, equipped with a liquid nitrogen dewar, which was put in a vacuum sleeve (p ~ 0.1 bar) and were gradually cooled to approximately 170 K over the course of 10 to 15 minutes (cooling rate of approximately 10 K min^{-1}) before being inserted into the transmission electron microscope (TEM) column, with data being collected at a final temperature of 98 K. 3DED data were collected using a JEOL JEM-2100 TEM operating at 200 kV, equipped with a Timepix detector from Amsterdam Scientific Instruments. The experiment was carried out using Instamatic,[1] and data reduction, as well as the merging of datasets collected from individual crystals, was performed using XDS.[2] The merged intensities were then used to solve the structures with SHELXT.[3] Reciprocal space visualizations were prepared using REDp.[4] All the data are available via Zenodo (DOI: 10.5281/zenodo.18029105), along with sped-up videos showing the diffraction frames of one representative dataset each for the studied ZIF-7 and ZIF-9 crystals.[5]

For each of the two samples, a total of four datasets were collected, integrated using XDS[2], and subsequently merged using XDSMERGE[2], and the merged intensities were used to solve the structure using SHELXT[3]. For a comparison of individual versus merged datasets, see Table S2. Using the merged data, all non-hydrogen atoms could be located in the initial structure solution for both the ZIF-7 and ZIF-9 samples. The unit cell parameters shown in Table S2 were taken from the first crystal measured and were used for the subsequent refinement. The refinement was done using SHELXL[6], with electron atomic scattering factors as published by Peng[7]. Hydrogen atoms were placed and constrained using HFIX/AFIX 43 commands, as only the hydrogen on the carbon in the ···N-C-N··· motif of the benzimidazolate molecule could reliably be located in the Fourier maps. All non-hydrogen atoms were refined with atomic anisotropic displacement parameters, with minor restraints applied using SIMU at default settings in SHELXL for activated ZIF-7, resulting in a total of 24 restraints for two groups of three atoms. Observed electrostatic potential maps of the two structures are shown in Figure S4.

S1.2 Powder X-ray diffraction (PXRD)

Initial PXRD data of as-synthesised and activated materials were collected to verify their purity and phase transformation upon solvent removal. A Siemens D5005 diffractometer in Bragg-Brentano geometry using CuKα (λ = 1.5418 Å) radiation in a 2θ range from 5° to 50° with a step size of 0.02° was used. All samples were finely ground and placed on a glass holder. Sample purity was confirmed by profile fits (Pawley method[8]) using the TOPAS academic v6 software package[9].

High-resolution PXRD patterns were collected for ZIF-7 and ZIF-9 activated materials loaded in capillaries at room temperature. The obtained patterns were refined against the crystal structures derived via 3DED using the Rietveld method. The PXRD pattern of ZIF-9 was collected at beamline 9 of DELTA (Dortmunder Elektronenspeicherring-Anlage, Dortmund, Germany) using a Dectris Pilatus 100K detector and a wavelength of 0.6199 Å. The PXRD pattern of ZIF-7 was

collected on a STOE STADI P diffractometer from 5° to 90° with a step size of 0.015° in transmission geometry using CuKα_1 radiation (1.540598 Å).

Variable temperature PXRD was performed at Beamline 9 of DELTA with a monochromatic X-ray beam (λ = 0.6199 Å) using a MAR345 image plate detector. The samples underwent a heating/cooling cycle. The investigated temperatures ranged from 21 °C to 494 °C and from 21 °C to 450 °C, for ZIF-9 and ZIF-7, respectively. Finely ground samples were sealed in quartz glass capillaries (outer diameter 0.5 mm) under N_2 atmosphere, placed on an Anton Paar DHS1100 hot stage and heated under a graphite dome. Temperature calibration of the hot stage was performed by reference variable temperature PXRD measurements of a silicon standard using the equation of state for silicon.[10] Profile fits of the scans were performed using the Pawley method.[8] For the np phases, the Stephens model[11] was applied to account for anisotropic peak broadening.

S1.3 Rietveld refinement of PXRD data

Rietveld refinements were carried out using the *TOPAS-academic v6* software package[9] and the routines implemented therein.

Structureless profile fitting (Pawley method) of the PXRD patterns of ZIF-9 and ZIF-7 provided the unit cell parameters, peak shape function parameters and the background curve, which were taken over into the Rietveld refinement and further refined later. The structural model extracted from 3DED was used as a starting point (without H-atoms) and refined applying soft restraints on the M–N distances (2 ± 0.05 Å) and the N–M–N angles (109.5° ± 10°) to maintain the tetrahedral coordination environment around the metal centres. All benzimidazolate linkers were treated as rigid bodies, which could be translated or rotated. Throughout the refinement, a Stephens model was applied to account for the strong anisotropic peak broadening present in the PXRD patterns.[11]

S1.4 Density functional theory (DFT) calculations

The geometry and lattice optimizations were performed employing dispersion-corrected periodic DFT calculations using the QUICKSTEP module of the CP2K code[12]. The gradient-corrected PBE functional[13] was used together with the dispersion correction "D3" by Grimme et al[14]. MOLOPT Double-ζ valence polarized Gaussian basis sets were employed for all atoms, and Goedecker–Teter–Hutter (GTH) type pseudopotentials were used to represent the electron-ion interactions.[15–17]. The Brillouin zone was sampled at the Γ point only due to the size of the systems. For similar porous systems, it was found to be crucial to use a sufficiently high plane-wave cutoff E_{cut}, since the computation of Coulomb and exchange-correlation energies is performed on a real-space grid[18]: A cutoff of E_{cut} = 2000 Ry and a relative cutoff E^{rel}_{cut} = 250 Ry was used throughout. For both the ***np*** and ***lp*** phases, lattice optimizations were performed in a primitive cell (174 atoms; six Zn(bim)$_2$ formula units) without any symmetry constraints (space group *P*1). The DFT optimized geometries are provided as CIF-files as Supporting Information.

Table S1. Total DFT energies (PBE+D3) and cell parameters of the optimized structures.

**Phase**	ZIF-7 ***op*** phase	ZIF-7 ***np*** phase
a / Å	**13.657**	10.440
b / Å	13.660	14.757
c / Å	13.658	14.377
α / °	104.98	114.09
β / °	104.92	106.53
γ / °	104.95	92.53
energy / Ha	–1115.408956	–1115.436108

S1.5 Thermogravimetric analysis (TGA)

Thermogravimetric analysis was performed with a TA Instruments SDT-650 instrument using alumina crucibles and a 10 °C min^{-1} heating rate up to 600 °C in nitrogen atmosphere (N_2 flow of approx. 100 mL/min).

S1.6 Differential scanning calorimetry (DSC)

A DSC 25 instrument (TA Instruments) was used to measure the phase transition temperatures and gravimetric transition enthalpies of the ZIFs. The temperature and cell constant were calibrated using an indium standard. 5-10 mg of ZIF samples were loaded in T-zero aluminum hermetic pans (TA Instruments) with a hole on their lids. An empty, hermetically sealed T-zero aluminum pan was used as a reference. Heating and cooling rates of 10 °C min^{-1} were used with a 50 mL/min N_2 flow into the sample cell and the following temperature profiles were applied: 50 °C → 535 °C → 200 °C → 535 °C → 200 °C → 535 °C → 200 °C for ZIF-9; 50 °C → 510 °C → 200 °C → 510 °C → 200 °C → 510 °C → 200 °C for ZIF-7.

Transition temperatures and transition enthalpies were determined using the TRIOS software from TA Instruments. Peaks of interest were analysed by selecting the appropriate temperature range. The lower and upper limits, as signalled by baseline deviation (lower limit) and return to baseline (upper limit), of the temperature range were chosen to encompass the phase transition signal. However, there were instances that return to the baseline was not monitored potentially due to the extension of the phase transition signal over the material's thermal decomposition (i.e. thermal decomposition starts close to completion of the thermally triggered ***np-lp*** transition).

A sigmoidal baseline was generated using the TRIOS software with tangent slopes in both bounds, when possible. Specifically, for the heating branches of both materials, a less physical zero slope (horizontal) was used in the upper bounds since the sample heating was limited to 510 °C (ZIF-7) or 535 °C (ZIF-9) to avoid decomposition.

The extrapolated onset temperatures were reported as transition temperatures, as it is standard in DSC analysis of solid-solid phase transformations due to the fact that peak temperatures are dependent on heating rate.[19,20]

S1.7 ^{1}H NMR spectroscopy

Approximately 10 mg of powder was digested in a solution of 0.1 mL of 35 wt.% DCl in D_2O and 0.6 mL of DMSO-d_6. ^{1}H NMR spectra were acquired on Bruker DPX300, DPX 500 or Agilent DD2 500 spectrometers.

S2 Materials synthesis

S2.1 ZIF-7

For ZIF-7 synthesis, the procedure described by He et al.[21] was adopted. $Zn(NO_3)_2 \cdot 6H_2O$ (0.9129 g, 3 mmol, 1 eq.) was dissolved in 10 mL EtOH and bimH (0.7218 g, 6 mmol, 2 eq.) was dissolved in 30 mL EtOH, to which 3.6 mL of NH_3 solution (25%) (3.3 g, 48 mmol, 16 eq.) was added. Both solutions were sonicated until complete dissolution. The Zn-solution was afterwards added to the bim-solution and the mixture was further stirred for 0.5 h. Subsequently, the suspension was vacuum-filtered through a glass frit funnel, washed with an excess of EtOH (ca. 100 mL) and filtered again. A portion of the obtained white powder was left overnight at room temperature (*ZIF-7 as-synthesised*) and further analysed. The remaining powder was transferred to a Schlenk tube and gradually ramped from ambient temperature to 200 °C over ca. 20 min using a silicone oil bath and heating plate (average heating rate of ca. 9 K min^{-1}) under dynamic vacuum (0.01 mbar). The sample was evacuated at 200 °C and 0.01 bar for 48 h and then allowed to cool down naturally to ambient temperature under vacuum to yield *ZIF-7 activated*. The activated sample was handled in air subsequently and remains in the ***np*** phase in the absence of penetrating solvents.

S2.2 ZIF-9

The synthetic procedure initially reported by Park et al.[22] and subsequently modified by Nguyen et al.[23] was followed.

$Co(NO_3)_2 \cdot 6H_2O$ (0.4210 g, 1.442 mmol, 1.4 eq.) and bimH (0.1205 g, 1.0200 mmol, 1 eq.) were dissolved in 36 mL DMF in a 100 mL glass vial. The reaction solution was sonicated until complete dissolution and subsequently heated for 48 h at 130 °C in an oven under air atmosphere sealed with a high-temperature screw cap. After cooling down to room temperature, the reaction solution was vacuum-filtered through a glass frit funnel and washed with excess of DMF (ca. 80 mL). Subsequently, the obtained solid solid mixture of two different kinds of crystals (ZIF-9 and cobalt formate)[24] was soaked in a vial with 30 mL chloroform and 1 mL DMF. As a result, the purple ZIF-9 crystals floated on the surface, while the red cobalt formate crystals remained at the bottom. After separation of the ZIF-9 crystals from the cobalt formate crystals, the ZIF-9 crystals were vacuum filtered again and either left overnight at room temperature (*ZIF-9 as-synthesised*) or placed in a Schlenk tube and gradually ramped from ambient temperature to 200 °C over ca. 20 min using a silicone oil bath and heating plate (average heating rate of ca. 9 K min^{-1}) under dynamic vacuum (0.01 mbar). The sample was evacuated at 200 °C and 0.01 bar for 48 h and then allowed to cool down naturally to ambient temperature under vacuum to yield *ZIF-9 activated*. The activated sample was handled in air subsequently and remains in the ***np*** phase in the absence of penetrating solvents.

S3 Materials characterization

S3.1 Laboratory PXRD data

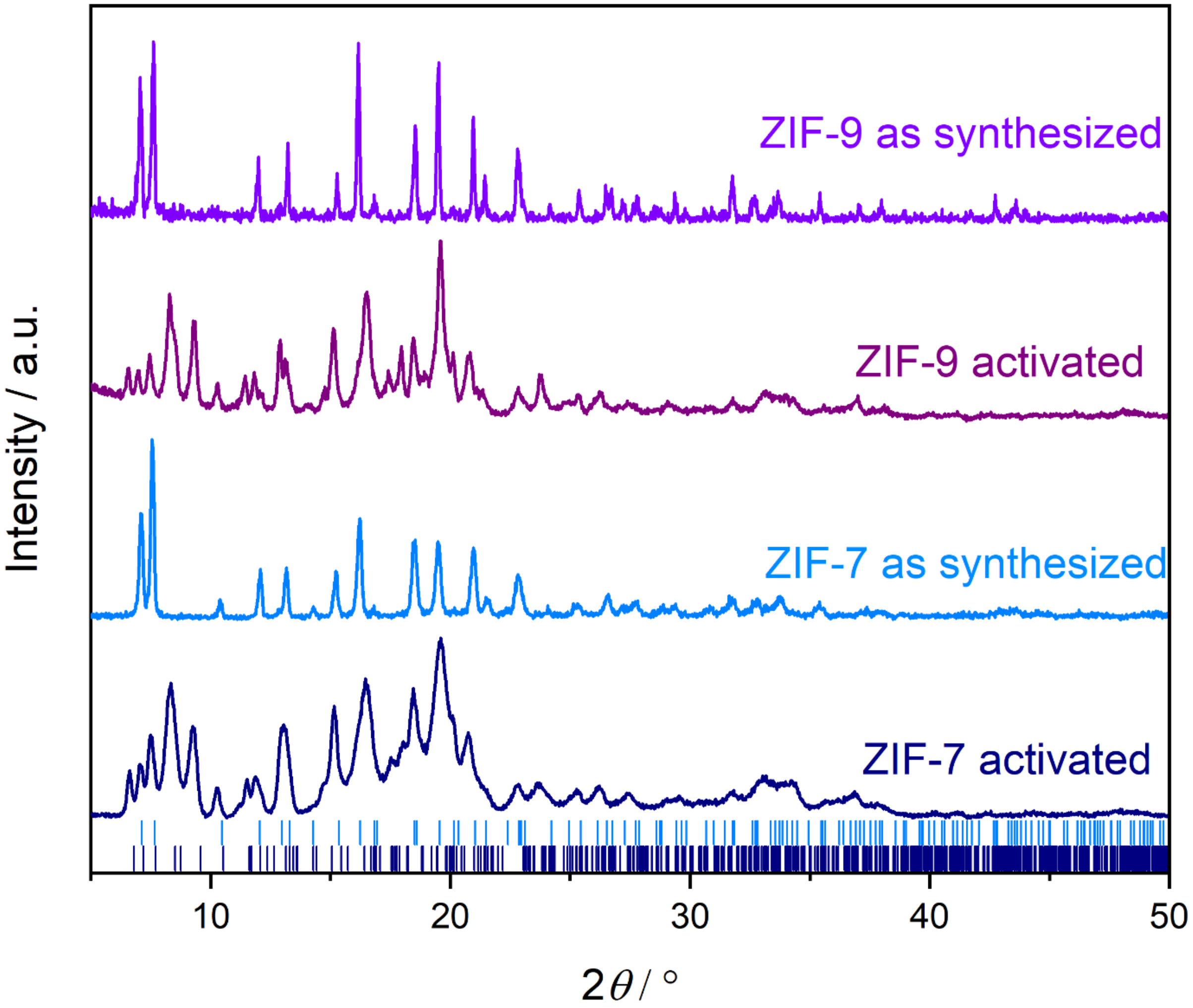

Figure S1. PXRD patterns of as-synthesised (***lp***) and activated (***np***) ZIF-9 and ZIF-7. Tick marks represent the characteristic Bragg reflection positions of ZIF-7 ***np*** phase (purple)[25] and ***lp*** phase (dark blue)[22]

S3.2 1H NMR spectra

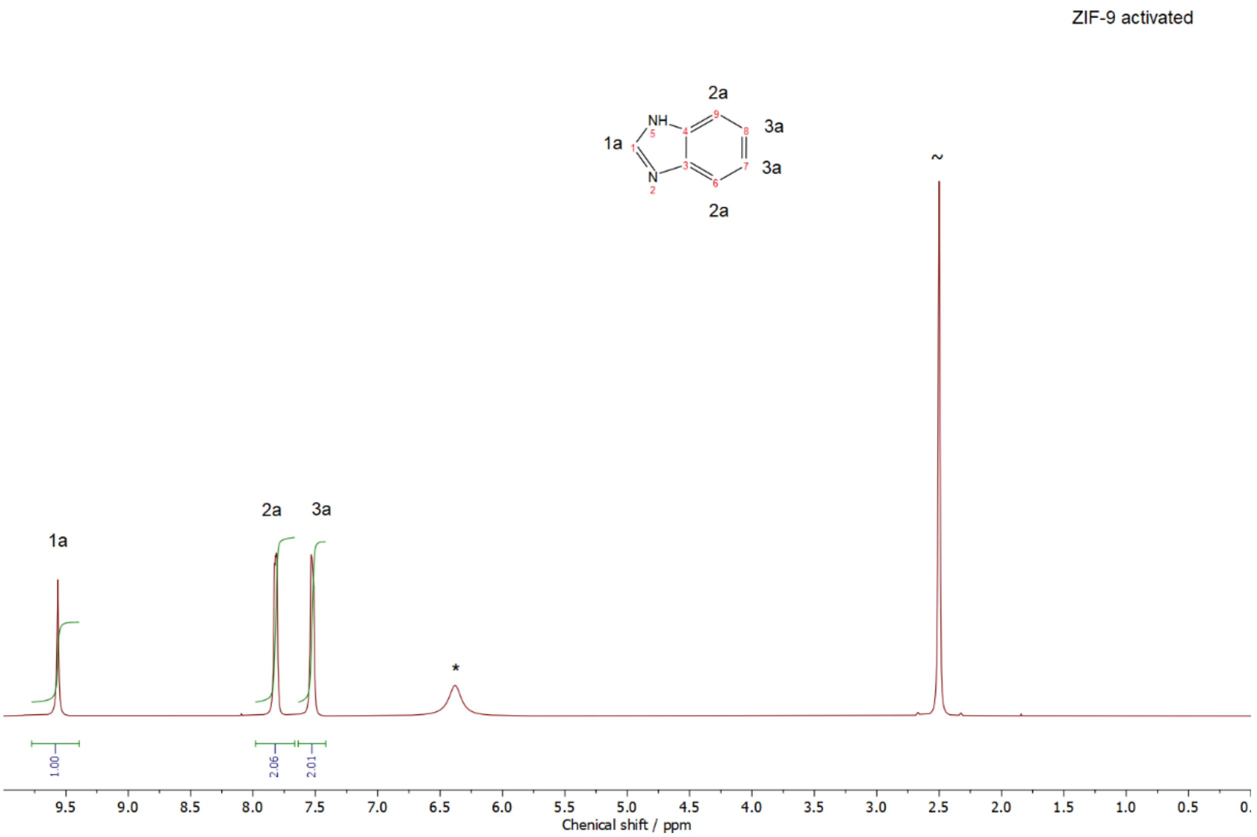

Figure S2. 1H NMR spectrum of a digested sample of activated ZIF-9. DMSO is marked with ~ and H_2O/HDO is marked with *.

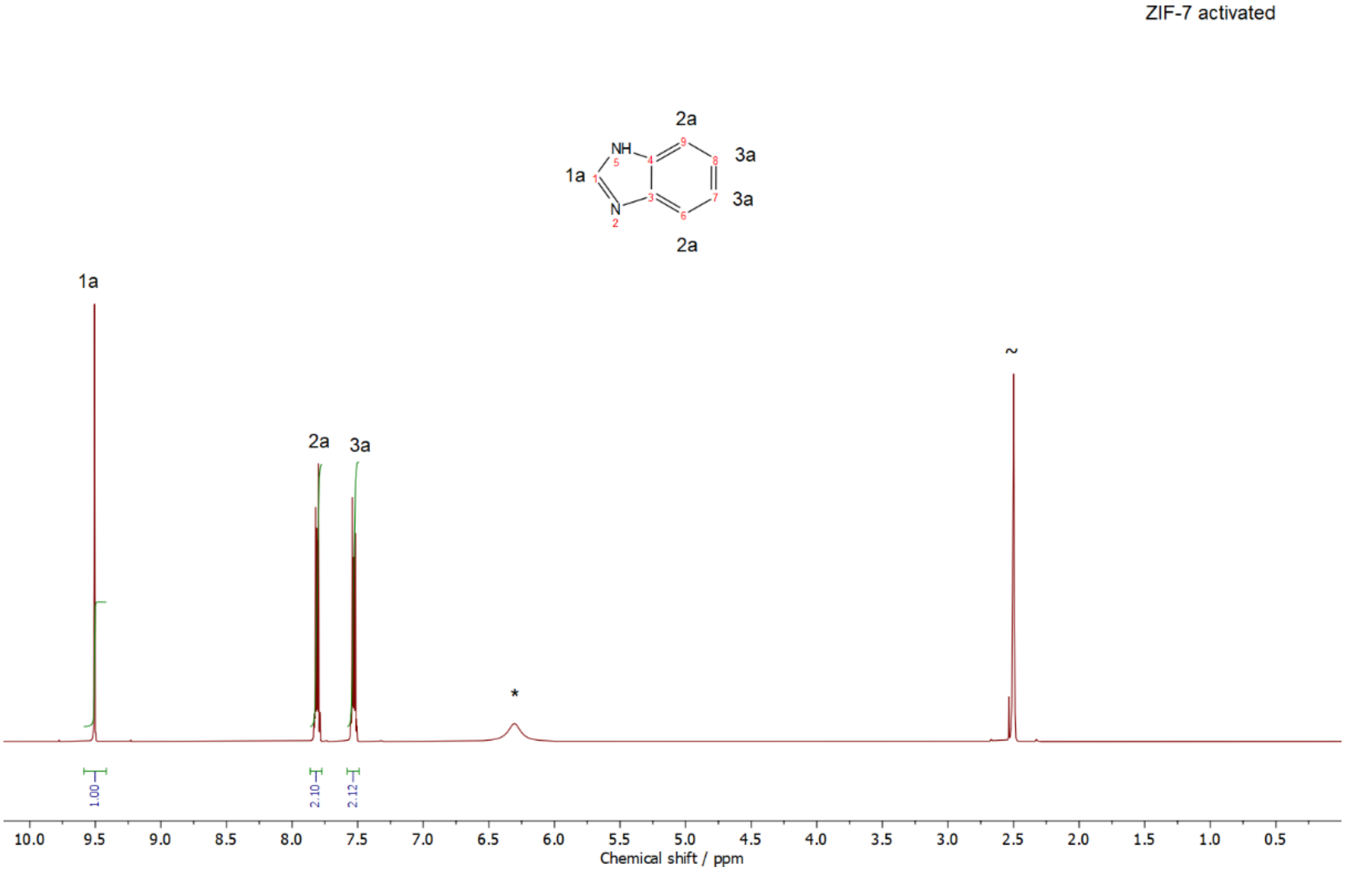

Figure S3. ^{1}H NMR spectrum of a digested sample of activated ZIF-7. DMSO is marked with ~ and H_2O/HDO is marked with *.

S3.3 3DED and Rietveld refinement data

Table S2. Crystallographic table for electron diffraction data of ZIF-7 (CCDC deposition code: 2493470) and ZIF-9 (CCDC deposition code: 2493472) activated phases.

Compound	ZIF-7 activated	ZIF-9 activated
Empirical formula	$C_{42}H_{30}N_{12}Zn_3$	$C_{42}H_{30}Co_3N_{12}$
Wavelength / Å	0.0251	0.0251
Crystal system	Triclinic	Triclinic
Space group	$P\bar{1}$	$P\bar{1}$
a / Å	11.710(2)	11.340(2)
b / Å	14.850(3)	14.680(3)
c / Å	14.930(3)	14.610(3)
α / °	111.08(3)	111.07(3)
β / °	108.69(3)	108.14(3)
γ / °	94.67(3)	94.47(3)
Volume / $Å^3$	2237.2(10)	2106.7(9)
Z	2	2
Index ranges	$-14 \le h \le 14$	$-14 \le h \le 14$
	$-18 \le k \le 18$	$-18 \le k \le 17$
	$-18 \le l \le 18$	$-17 \le l \le 18$
Reflections collected	24989	23530
Independent reflections	7353	7572
	[R(int) = 0.2117]	[R(int) = 0.1783]
Completeness (to 0.8 Å resolution)	82 %	88 %
R_1 (ED model) [$I > 2\sigma(I)$]	0.1661	0.1501
Merging information		
Individual datasets merged/collected	4/4	4/4
Average completeness (to 0.8 Å resolution)	44(3)	45(3)
Average R_1 for individual datasets ([$I > 2\sigma(I)$])	19(3)	18(1)
Average no. of non-positive definite ADPs	13(7)	5(4)

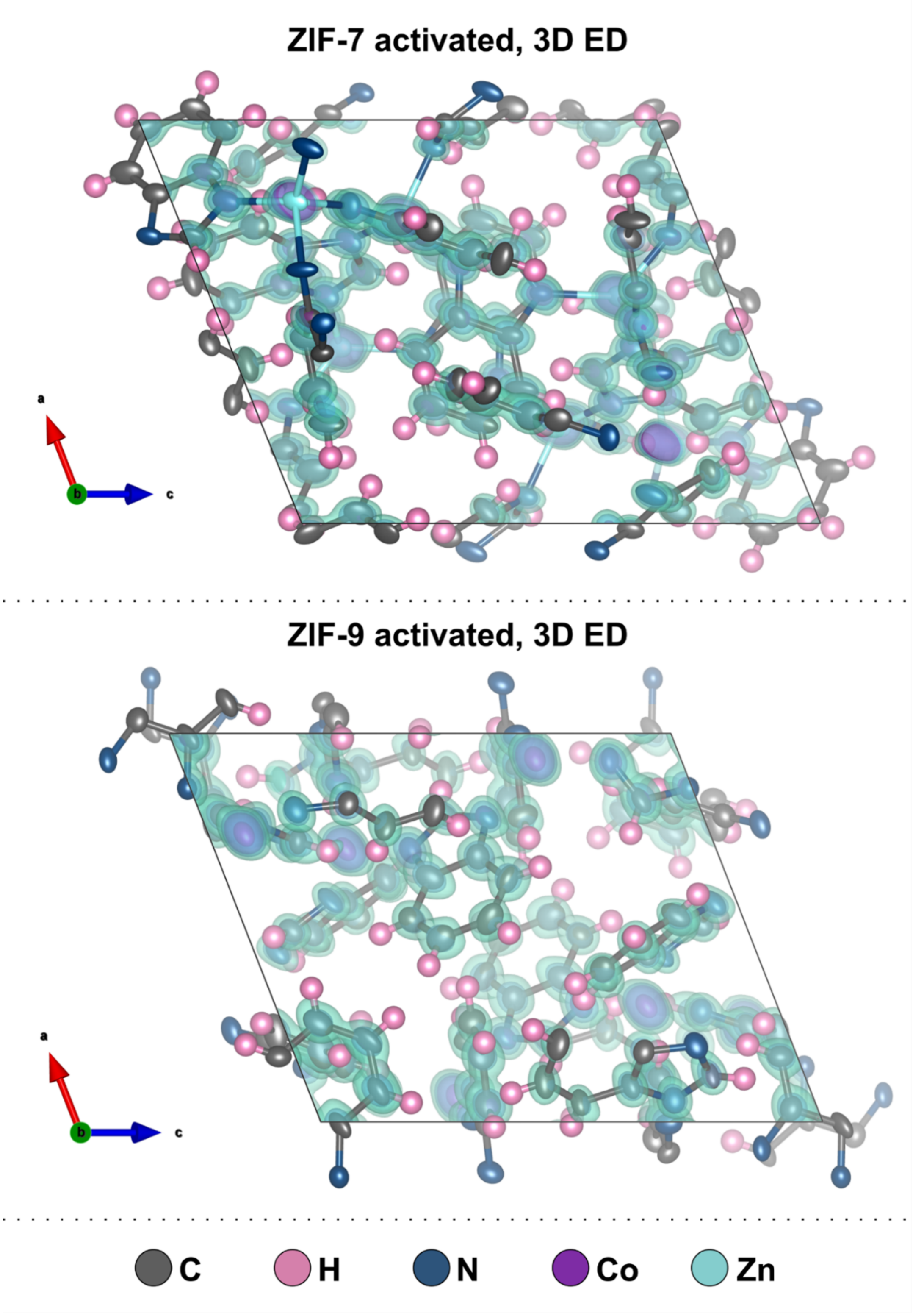

Figure S4. Structures of activated ZIF-7 and ZIF-9 as refined against 3DED data, with three isosurface levels of observed electrostatic potential. Highest to lowest: purple, teal, pink, corresponding to approximately 16, 8, and 4 σ. Atomic anisotropic displacement parameters are drawn at the 50% probability level.

Table S3. Crystallographic data for the Rietveld refinement of the activated ZIF-7 (CCDC deposition code: 2493469) and ZIF-9 (CCDC deposition code: 2493471) phases.

Compound	ZIF-7 activated	ZIF-9 activated
wavelength / Å	1.5406	0.6199
chemical formula	$C_{42}H_{30}N_{12}Zn_3$	$C_{42}H_{30}Co_3N_{12}$
crystal system	triclinic	triclinic
space group	$P\bar{1}$	$P\bar{1}$
a / Å	11.5640(18)	11.3961(17)
b / Å	14.4027(14)	14.3123(13)
c / Å	14.2930(16)	14.2295(16)
α / °	109.799(10)	109.831(10)
β / °	109.889(11)	109.636(9)
γ / °	96.067(9)	95.513(9)
V / Å^3	2039.5(5)	1997.9(5)
R_{Bragg} / %	1.21	1.36
R_{wp} / %	2.33	3.89
R_{exp} / %	0.96	20.83
χ	2.42	0.19

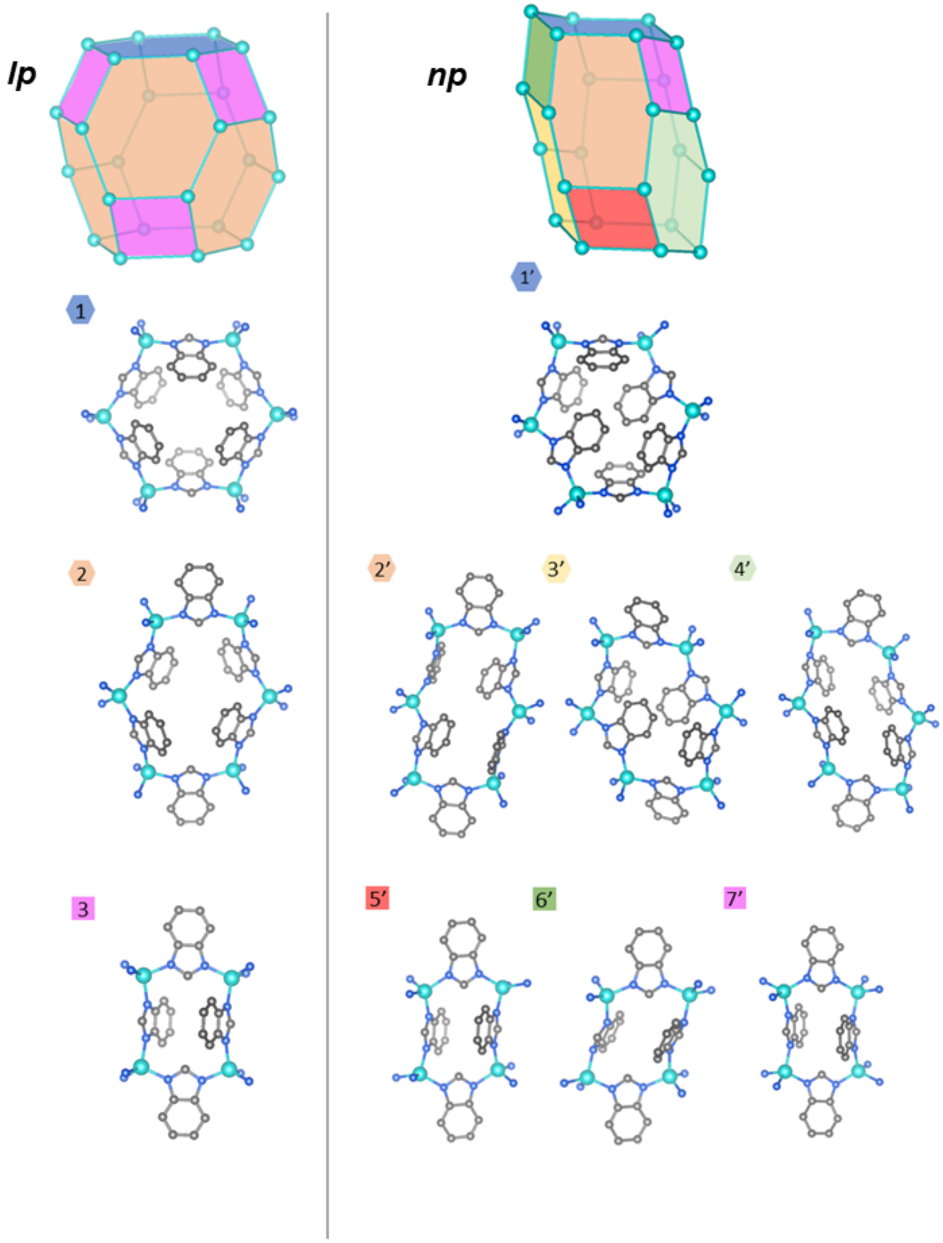

Figure S5. Structural representations of ZIF-7 ***np*** (right) sodalite-like cages and the unique four-membered rings and six-membered rings in comparison to ***lp*** phases[22] (CCDC: VEJZEQ) displayed on the left. Zn, teal; N, blue; C, grey; Hydrogen atoms are omitted for clarity.

S3.4 Hirshfeld surface analysis of DFT-optimized structures

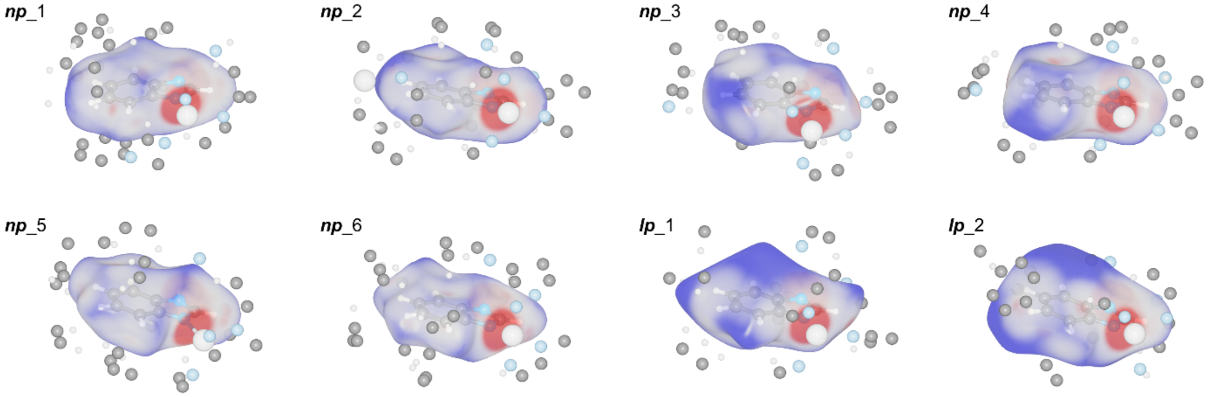

Figure S6: Hirshfeld surfaces mapped with d_{norm} (colour scale: –0.400, red, to 1.300, blue) for the crystallographically distinct bim^{-} linkers in guest-free ZIF-7, shown for the ***np*** phase (linkers 1-6) and the ***lp*** phase (linkers 1-2). Surfaces were generated in CrystalExplorer (version 2025).[26] The analysis is based on the DFT-optimised crystal structures. The parameter d_{norm} is the normalised contact distance, defined as the sum of internal (d_i) and external (d_e) distances from the Hirshfeld surface to the nearest atomic nuclei, each normalised by the respective van der Waals radii. Contacts shorter than the sum of the van der Waals radii appear in red, contacts close to the van der Waals separation in white, and contacts longer than the van der Waals separation in blue. Overall, the ***lp*** phase exhibits fewer short inter-linker contacts than the ***np*** phase, consistent with a less dense packing of the linkers. In addition to the intraframework Zn–N coordination bonds present in both phases, red patches highlight short non-bonded inter-linker contacts, including C–H$\cdots\pi$ interactions, which are more prominent in the ***np*** phase than in the ***lp*** phase.

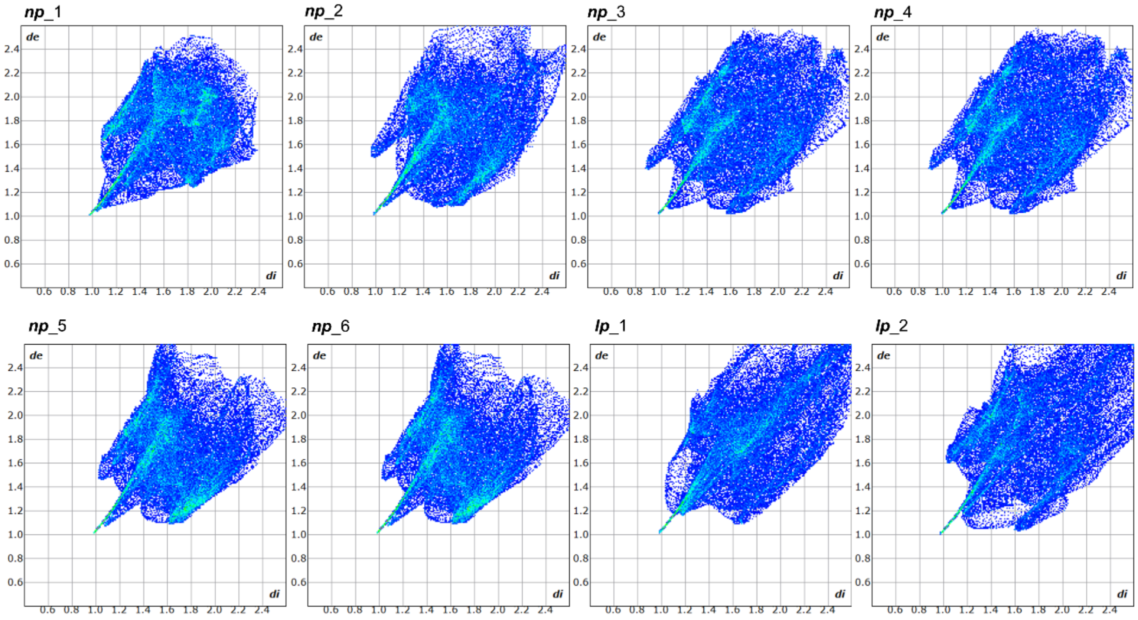

Figure S7: Two-dimensional Hirshfeld fingerprint plots (d_e vs d_i in Å) corresponding to the d_{norm}-mapped Hirshfeld surfaces shown in Figure S6, generated for each crystallographically distinct bim^{-} linker of guest-free ZIF-7 (***np*** phase: linkers 1–6; ***lp*** phase: linkers 1–2). The colour scale represents the relative frequency of surface points (blue = low, green = high). Compared to the ***lp*** phase, the fingerprint plots of the ***np*** phase show an increased intensity at shorter d_e and d_i values, consistent with a higher abundance of short inter-linker (non-bonded) contacts in the compact ***np*** phase.

Table S4: Decomposition of Hirshfeld surfaces into contributions from specific interatomic contact types. Values are given as percentages of the Hirshfeld surface area. The contact-type percentages primarily reflect the surface atom composition and packing topology and therefore do not directly quantify contact distances. The relative prevalence of short contacts is instead assessed from d_{norm}-mapped surfaces and the corresponding fingerprint plots (Figures S6 and S7).

Linker	H···H	C···H	C···C	Zn···N	Zn···H	N···H
np_1	38.0	32.1	4.6	9.8	1.3	13.0
np_2	42.7	30.0	0.7	7.8	2.1	14.0
np_3	37.9	34.5	0.7	7.4	1.5	15.9
np_4	37.9	34.4	0.7	7.5	1.5	16.0
np_5	42.4	31.1	1.0	8.5	2.8	14.0
np_6	42.5	31.1	1.0	8.5	2.8	13.9
lp_1	45.9	20.9	9.0	7.3	1.3	13.7
lp_2	40.6	32.4	2.3	9.0	2.1	12.5

S3.5 TGA data

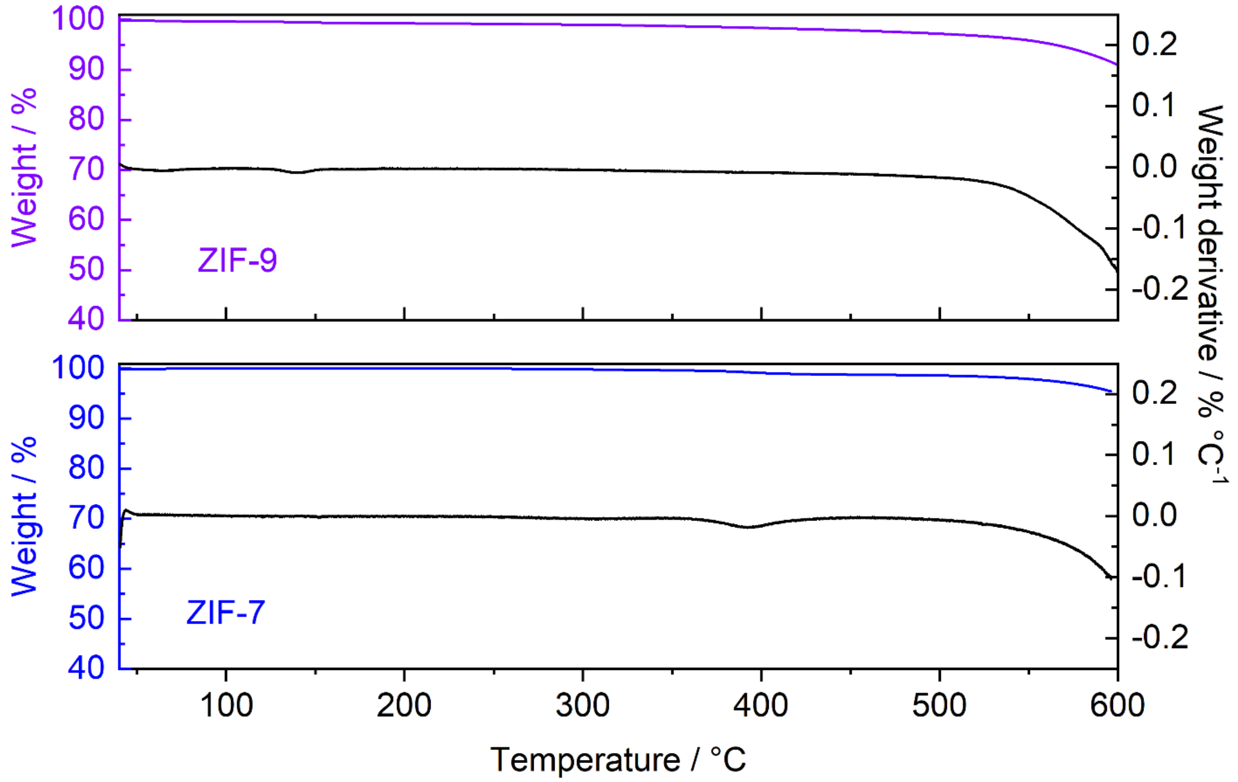

Figure S8. TGA data of ZIF-9 (top) and ZIF-7 (bottom). The coloured curves are weight vs temperature and the black curves are the weight derivative with respect to temperature vs temperature.

S3.6 DSC and VT-PXRD data

Table S5. Comparison of the phase transition temperatures $T_{np\text{-}lp}$ and $T_{lp\text{-}np}$, as well as the respective phase transition enthalpies $\Delta H_{np\text{-}lp}$ and $\Delta H_{lp\text{-}np}$ obtained from the DSC measurements. The molar enthalpies are referenced on one $M(bim)_2$ formula unit. The ***np-lp*** transition is observed at the heating branch, and the ***lp-np*** transition is observed on the cooling branch. Note that, except for the 1st heating cycle, no prominent peaks ascribed to structural transitions are observed for the subsequent branches of ZIF-7 material. Hence, the obtained values are significantly diminished.

**Compound**	**Cycle**	**Heating branch *np-lp***		**Cooling branch *lp-np***	
		$T_{np\text{-}lp}$ / °C	$\Delta H_{np\text{-}lp}$ / kJ mol^{-1}	$T_{lp\text{-}np}$ / °C	$\Delta H_{lp\text{-}np}$ / kJ mol^{-1}
**ZIF-9**	1	512	1.57	466	1.28
	2	516	0.72	464	1.01
	3	520	0.33	462	0.68
**ZIF-7**	1	471	1.62	384	0.20
	2	434	0.49	383	0.19
	3	429	0.28	381	0.19

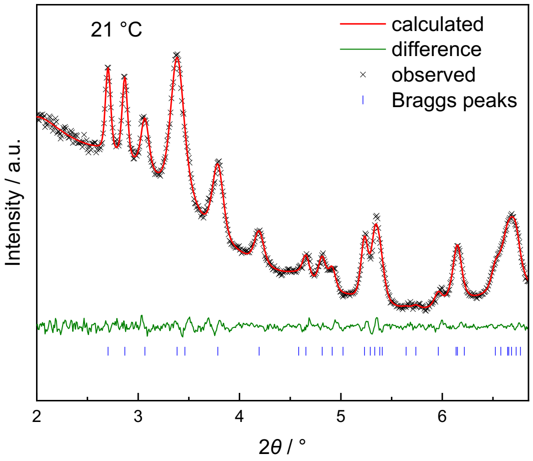

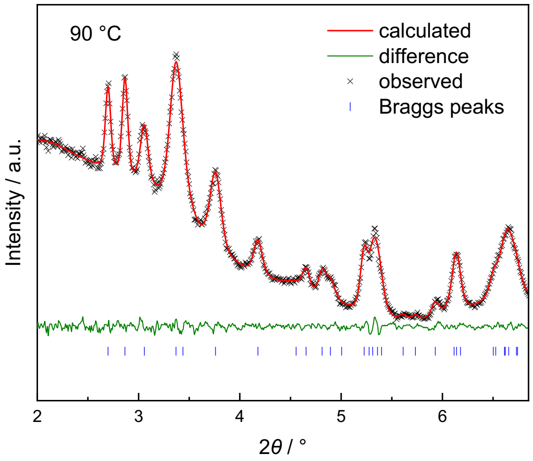

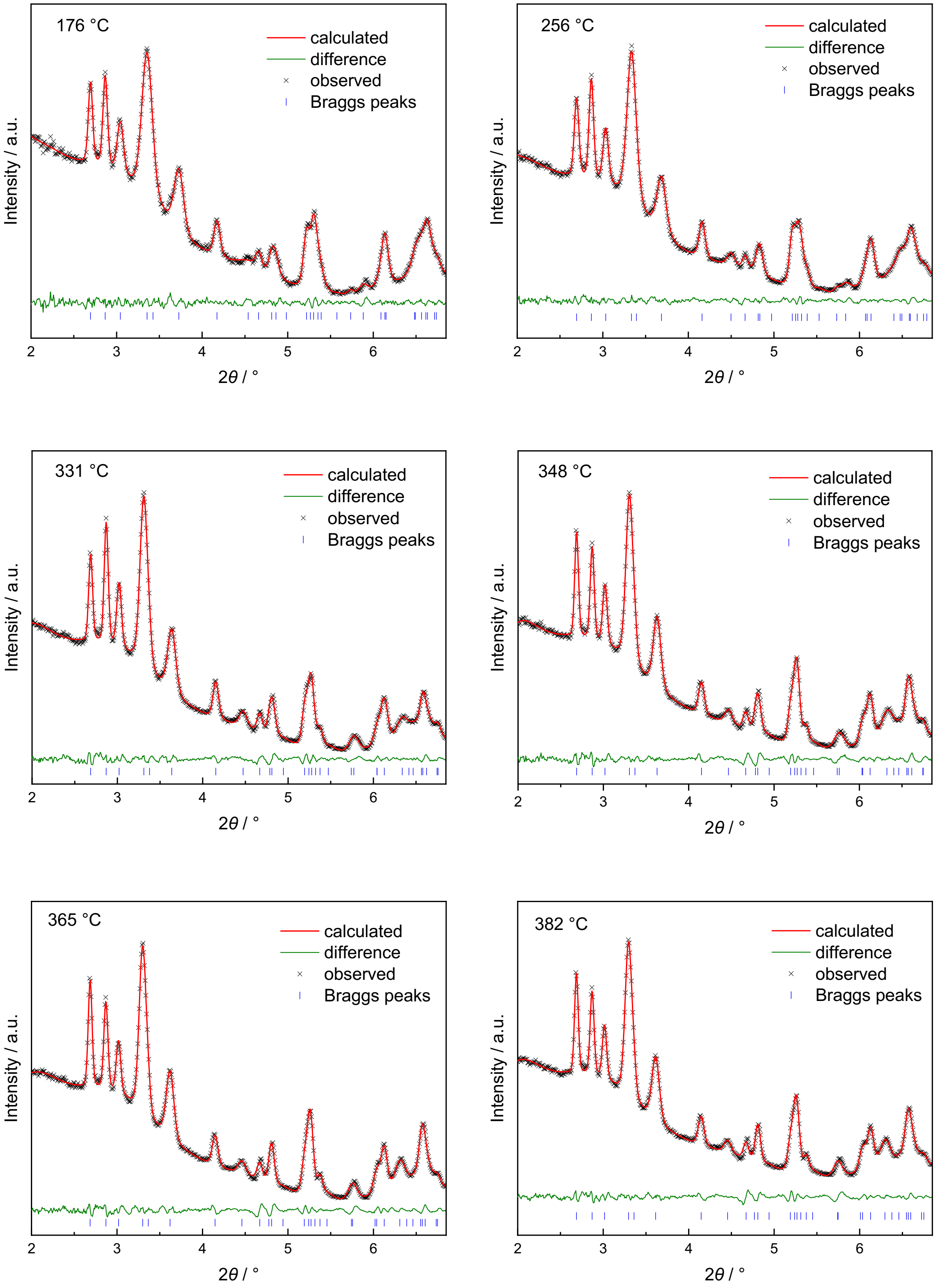
176 °C
256 °C
331 °C
348 °C
365 °C
382 °C
calculated
difference
observed
Braggs peaks
Intensity / a.u.
2θ / °
2
3
4
5
6

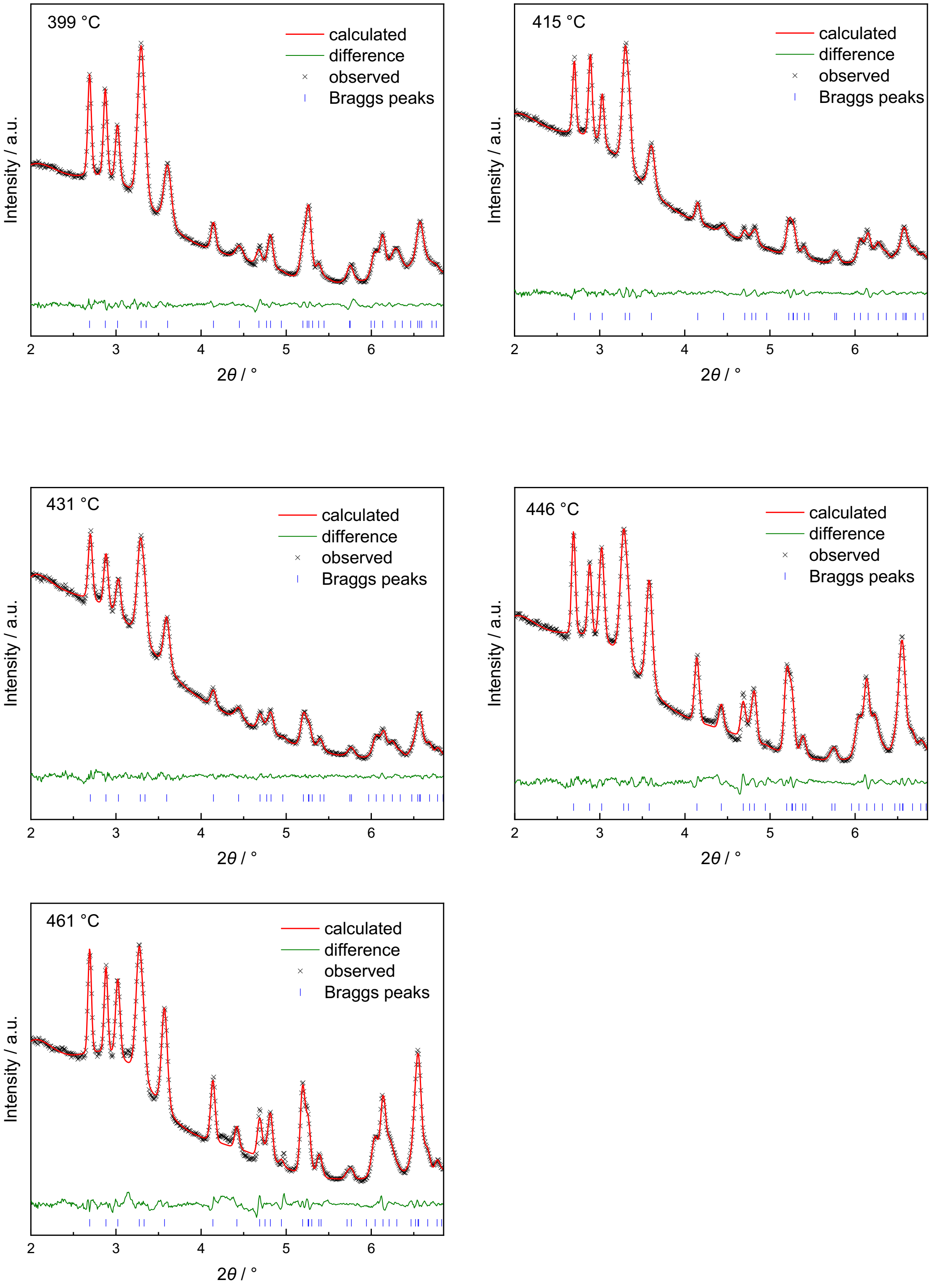

Figure S9. PXRD patterns with profile fits (Pawley method) of the ***np*** phase ZIF-9 during heating from 21 °C to 461 °C. The data exceeding the 2*θ* value of 6.87° were excluded due to the presence of reflections attributed to the graphite dome of the hot stage (see Figure S15).

Table S6. Crystallographic data for the ***np*** phase of ZIF-9 (space group $P\bar{1}$) determined via profile refinement of the VT-PXRD data collected in the range from 21 °C to 461 °C.

Temperature / °C	a / Å	b / Å	c / Å	α / °	β / °	γ / °	V / Å³	R_{wp}	R_{exp}	χ
**21**	11.495(16)	14.380(12)	14.322(13)	109.64(12)	109.74(14)	95.79(8)	2036(5)	0.93	105.21	0.01
**90**	11.572(15)	14.426(7)	14.369(19)	109.65(8)	109.9(2)	96.03(7)	2058(6)	0.86	109.05	0.01
**176**	11.636(15)	14.489(11)	14.383(13)	109.77(9)	109.78(14)	96.45(8)	2076(5)	0.85	112.87	0.01
**256**	11.762(0)	14.534(4)	14.430(9)	109.68(4)	110.00(9)	96.97(6)	2103(3)	0.71	121.39	0.01
**331**	11.869(9)	14.629(5)	14.422(9)	109.78(5)	109.71(8)	97.61(6)	2130(3)	0.93	129.91	0.01
**348**	11.896(9)	14.638(4)	14.432(10)	109.76(4)	109.78(10)	97.68(6)	2136(3)	0.96	128.15	0.01
**365**	11.921(12)	14.645(0)	14.432(12)	109.75(8)	109.78(11)	97.79(8)	2141(4)	0.98	128.29	0.01
**382**	11.987(17)	14.633(16)	14.462(18)	109.66(13)	110.02(16)	97.81(14)	2152(6)	1.45	129.01	0.01
**399**	11.970(9)	14.645(4)	14.430(9)	109.72(4)	109.88(9)	98.01(5)	2145(3)	0.84	128.86	0.01
**415**	11.980(8)	14.589(4)	14.381(6)	109.69(3)	110.12(6)	98.07(5)	2126(2)	0.71	115.69	0.01
**431**	11.943(12)	14.651(5)	14.440(11)	109.77(5)	109.79(11)	97.88(9)	2145(4)	0.99	129.01	0.01
**446**	12.058(5)	14.653(4)	14.416(5)	109.54(3)	110.04(4)	98.35(3)	2156.0(16)	1.07	112.84	0.01
**461**	12.093(5)	14.661(4)	14.409(6)	109.51(3)	110.03(6)	98.50(2)	2160.8(2)	1.25	115.21	0.01

Table S7. Thermal expansion of the ZIF-9 ***np*** phase represented by the expansion of orthogonal principal axes, as determined by PAScal.[27]

Temperature / °C	x_1 / % change	x_2 / % change	x_3 / % change	V / Å³
**21**	0	0	0	2036.3376
**90**	-0.0426	0.3302	0.7664	2057.8489
**176**	-0.1837	0.5527	1.5598	2075.6989
**256**	-0.3763	0.8539	2.8003	2103.1928
**331**	-0.445	0.9712	4.0935	2130.6599
**348**	-0.4687	1.0502	4.3218	2136.4886
**365**	-0.5107	1.0596	4.5674	2140.785
**382**	-0.5127	1.197	4.984	2152.2356
**399**	-0.7329	1.0559	5.0278	2145.108
**415**	-1.2927	0.7576	5.0541	2126.6609
**431**	-0.5689	1.1182	4.7798	2145.0796
**446**	-0.8899	0.9514	5.8368	2155.7729
**461**	-0.9395	0.9199	6.1762	2160.886

Table S8. Normalized components of the principal axes projected onto the crystallographic axes of the ***np*** phase of ZIF-9 and the coefficient of thermal expansion along each of them. The thermal expansion coefficients were obtained through the linear fit of the principal axes parameters using PAScal.[27]

Axes	Component			α / MK⁻¹
	a	b	c	
**X_1**	0.5314	0.5653	0.6309	-22.044
**X_2**	-0.3616	-0.5598	0.7455	21.6487
**X_3**	0.9148	-0.4019	0.0387	137.6666

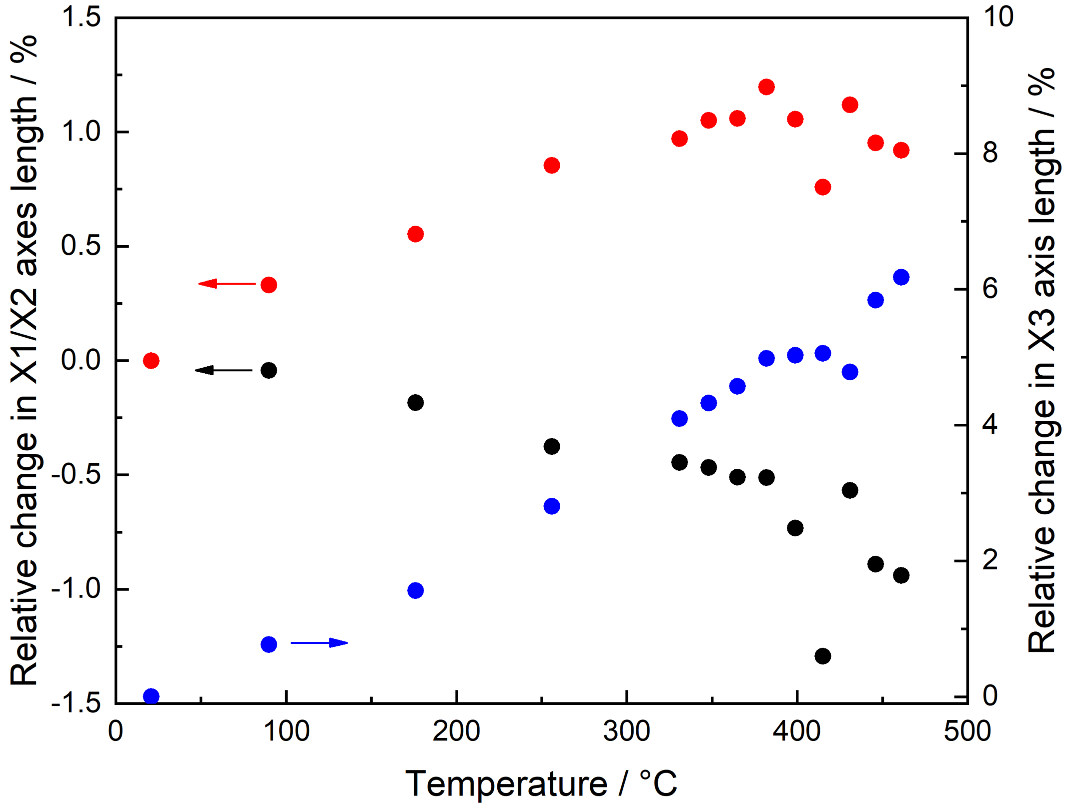

Figure S10. Temperature dependence of the length of the principal axes X_1 (black), X_2 (red), X_3 (blue) for the ***np*** phase of ZIF-9.

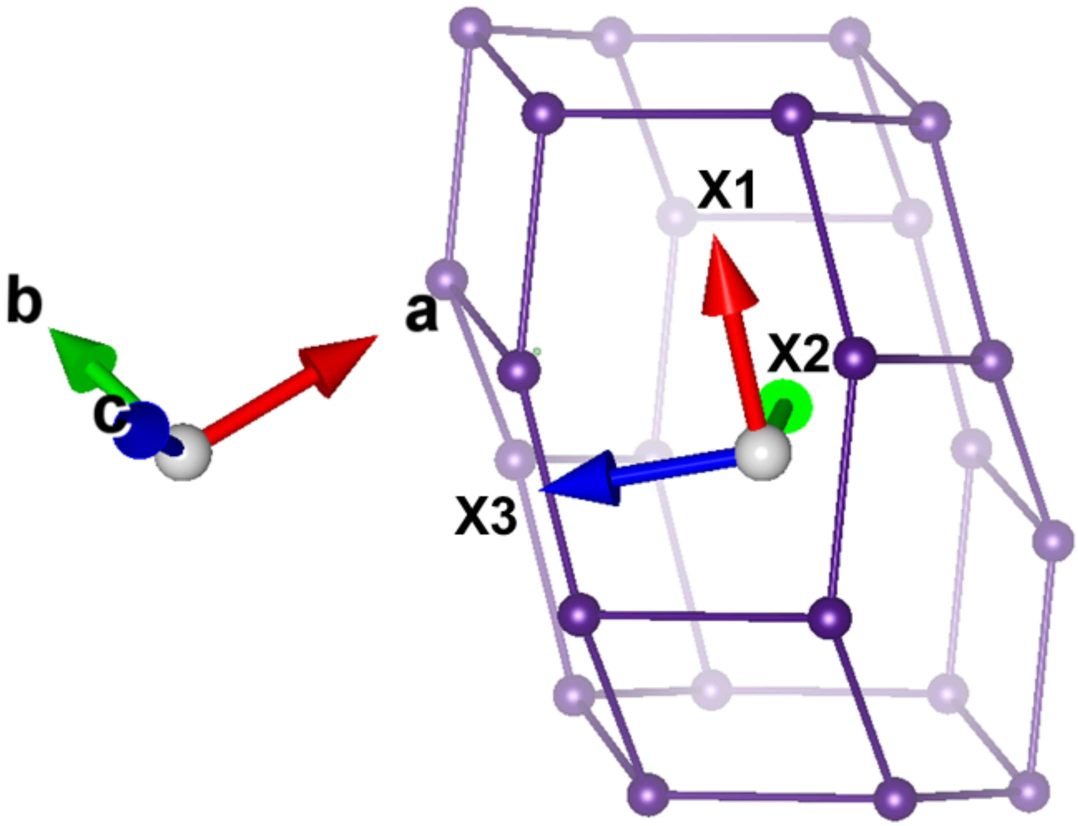

Figure S11. Representation of the sodalite-like cage of the ***np*** phase of ZIF-9 and the orientation of the orthogonal principal axes relative to the crystallographic axes.

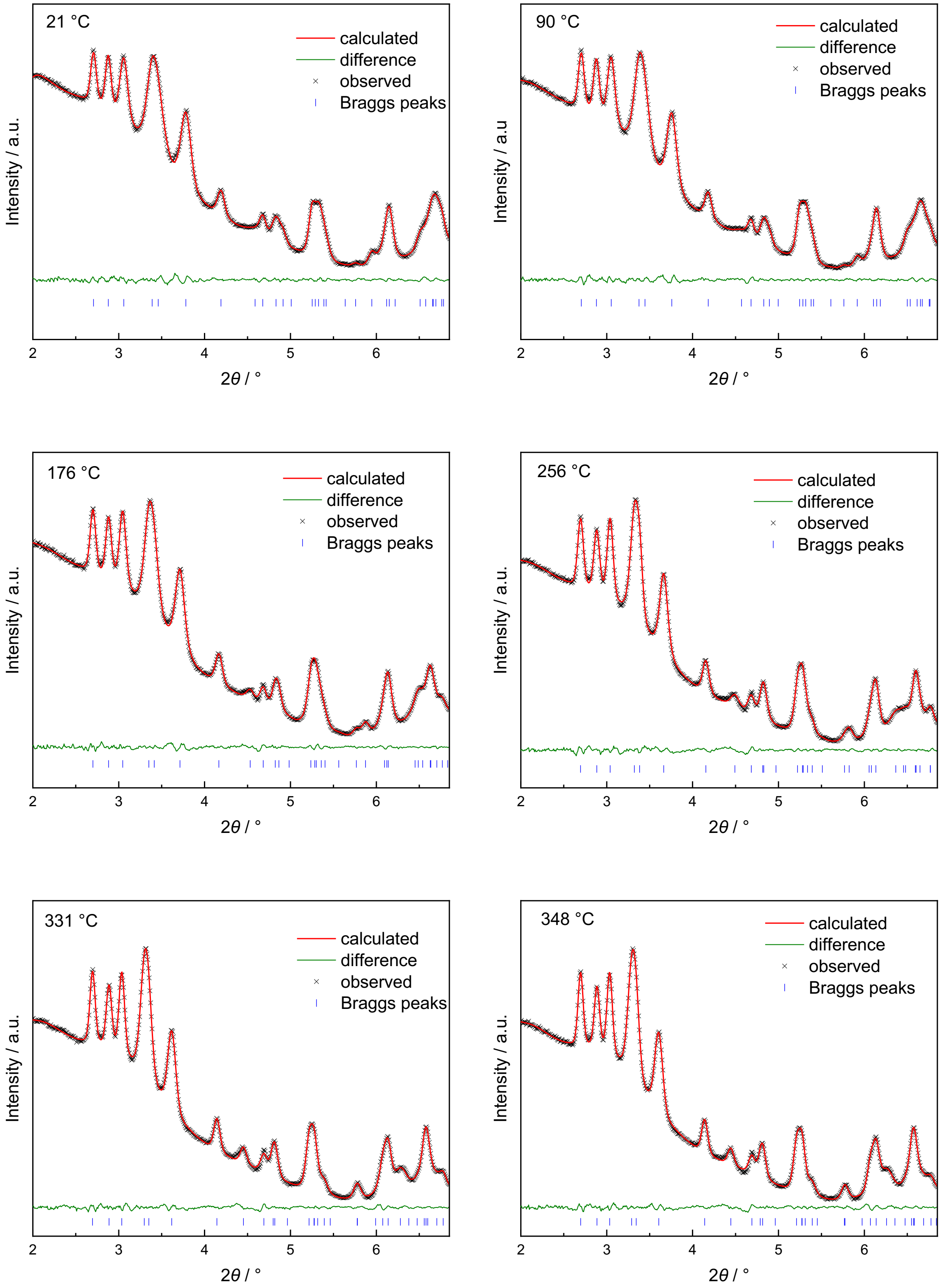
21 °C
calculated
difference
observed
Braggs peaks
Intensity / a.u.
2θ / °
90 °C
calculated
difference
observed
Braggs peaks
Intensity / a.u
2θ / °
176 °C
calculated
difference
observed
Braggs peaks
Intensity / a.u.
2θ / °
256 °C
calculated
difference
observed
Braggs peaks
Intensity / a.u.
2θ / °
331 °C
calculated
difference
observed
Braggs peaks
Intensity / a.u.
2θ / °
348 °C
calculated
difference
observed
Braggs peaks
Intensity / a.u.
2θ / °

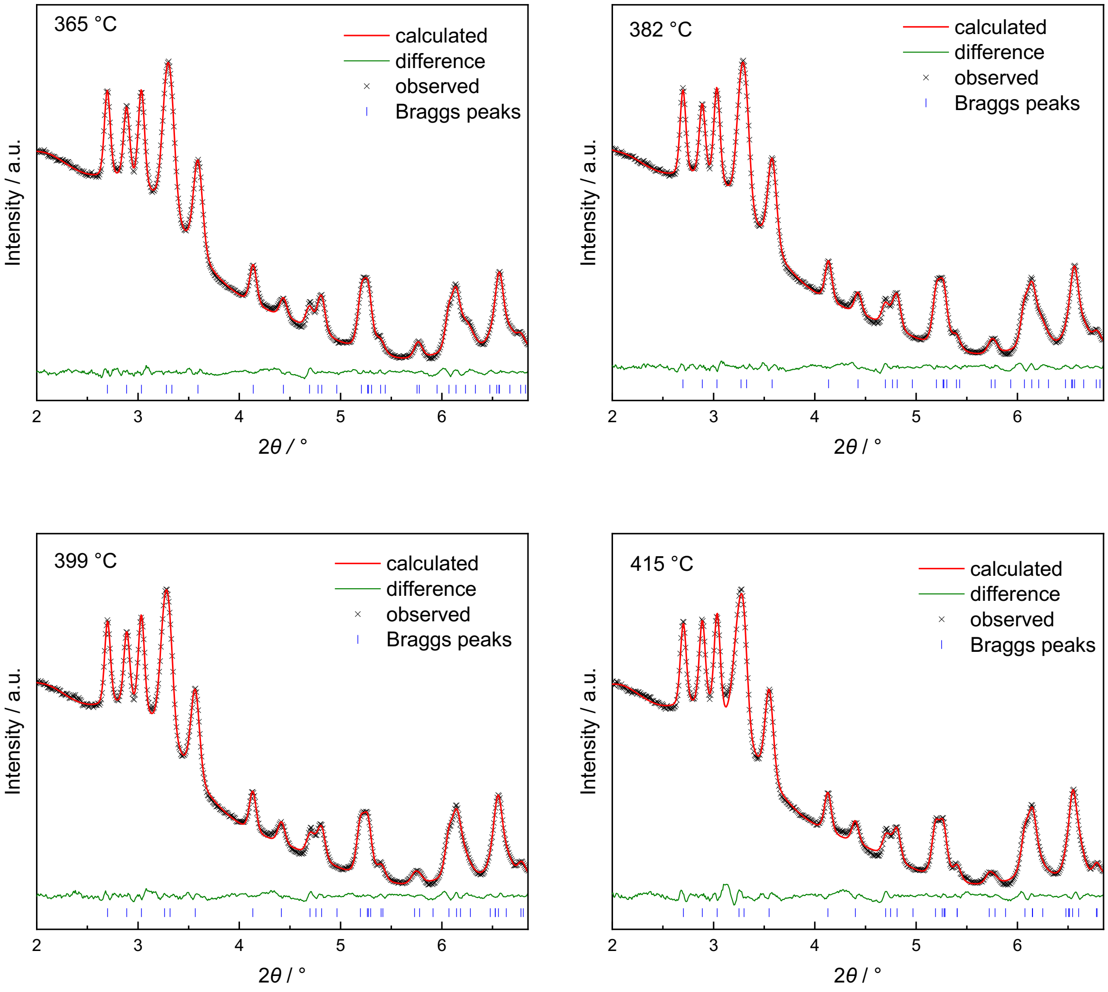

Figure S12. PXRD patterns with profile fits (Pawley method) of the ***np*** phase of ZIF-7 during heating from 21 to 415 °C. The data exceeding the 2θ value of 6.87° were excluded due to the presence of reflections attributed to the graphite dome of the hot stage (see Figure S17).

Table S9. Crystallographic data for the ***np*** phase of ZIF-7 (space group $P\overline{1}$) determined via profile refinement of the VT-PXRD data collected in the range from 21 °C to 415 °C.

Temperature / °C	a / Å	b / Å	c / Å	α / °	β / °	γ / °	V / Å³	R_{wp}	R_{exp}	χ
**21**	11.493(11)	14.404(7)	14.323(14)	109.94(6)	109.82(15)	95.86(5)	2032(4)	0.41	96.85	0.004
**90**	11.558(12)	14.442(10)	14.334(14)	109.97(7)	109.85(13)	96.11(5)	2047(4)	0.39	97.85	0.004
**176**	11.691(6)	14.490(4)	14.351(9)	109.84(4)	110.02(8)	96.60(4)	2074(2)	0.39	100.27	0.004
**256**	11.832(6)	14.547(6)	14.364(5)	109.70(3)	110.08(4)	97.20(3)	2103(2)	0.44	104.4	0.004
**331**	11.976(7)	14.584(4)	14.379(6)	109.53(3)	110.25(6)	97.80(3)	2129(2)	0.43	106.76	0.004
**348**	12.014(8)	14.592(4)	14.385(6)	109.50(3)	110.23(6)	97.95(4)	2137(2)	0.44	107.47	0.004
**365**	12.062(5)	14.597(5)	14.388(6)	109.46(4)	110.26(6)	98.12(3)	2144(2)	0.5	108.12	0.005
**382**	12.106(8)	14.602(4)	14.387(6)	109.43(3)	110.26(5)	98.30(4)	2151(2)	0.52	108.34	0.005
**398**	12.155(12)	14.605(7)	14.388(8)	109.35(4)	110.29(7)	98.49(6)	2159(3)	0.57	108.89	0.01
**415**	12.223(10)	14.597(6)	14.398(9)	109.20(5)	110.42(7)	98.68(5)	2169(3)	0.75	109.06	0.01

Table S10. Thermal expansion of the ZIF-7 ***np*** phase represented by the expansion of orthogonal principal axes, as determined by PAScal.[27]

Temperature / °C	x_1 / % change	x_2 / % change	x_3 / % change	V / Å³
**21**	0	0	0	2032.3726
**90**	-0.0995	0.1665	0.6589	2047.1307
**176**	-0.2227	0.3108	1.9522	2073.8395
**256**	-0.2999	0.422	3.3678	2103.3302
**331**	-0.4869	0.4696	4.7824	2128.958
**348**	-0.4772	0.4829	5.1416	2136.7693
**365**	-0.534	0.4808	5.5826	2144.4214
**382**	-0.6023	0.4634	5.9994	2150.9868
**398**	-0.635	0.4235	6.4557	2158.636
**415**	-0.6571	0.3665	7.0403	2168.6996

Table S11. Normalized components of the principal axes projected onto the crystallographic axes of the ***np*** phase of ZIF-7 and the coefficient of thermal expansion along each of them.

Axes	Component			α / MK^{-1}
	a	b	c	
**X_1**	0.5192	0.5285	0.6717	-16.6547
**X_2**	0.2748	0.7272	-0.629	10.3027
**X_3**	0.9416	-0.3342	-0.0409	178.2013

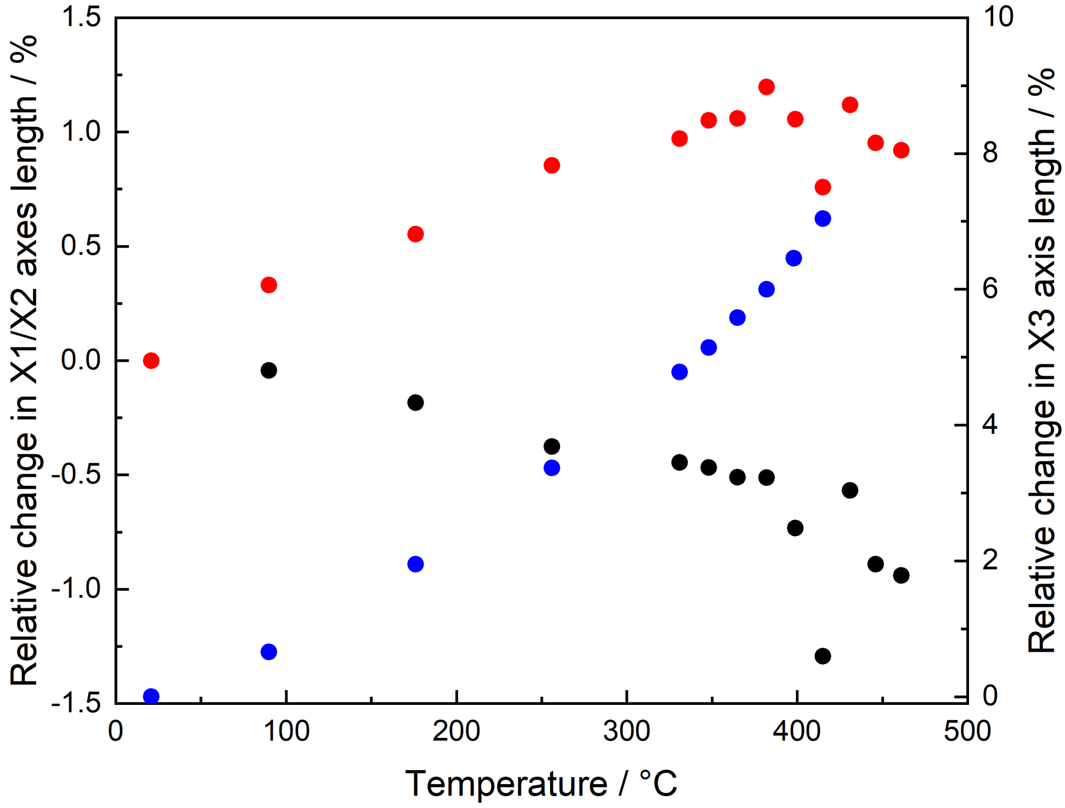

Figure S13. Temperature dependence of X_1 (black), X_2 (red), X_3 (blue) axis lengths for the ***np*** phase of ZIF-7.

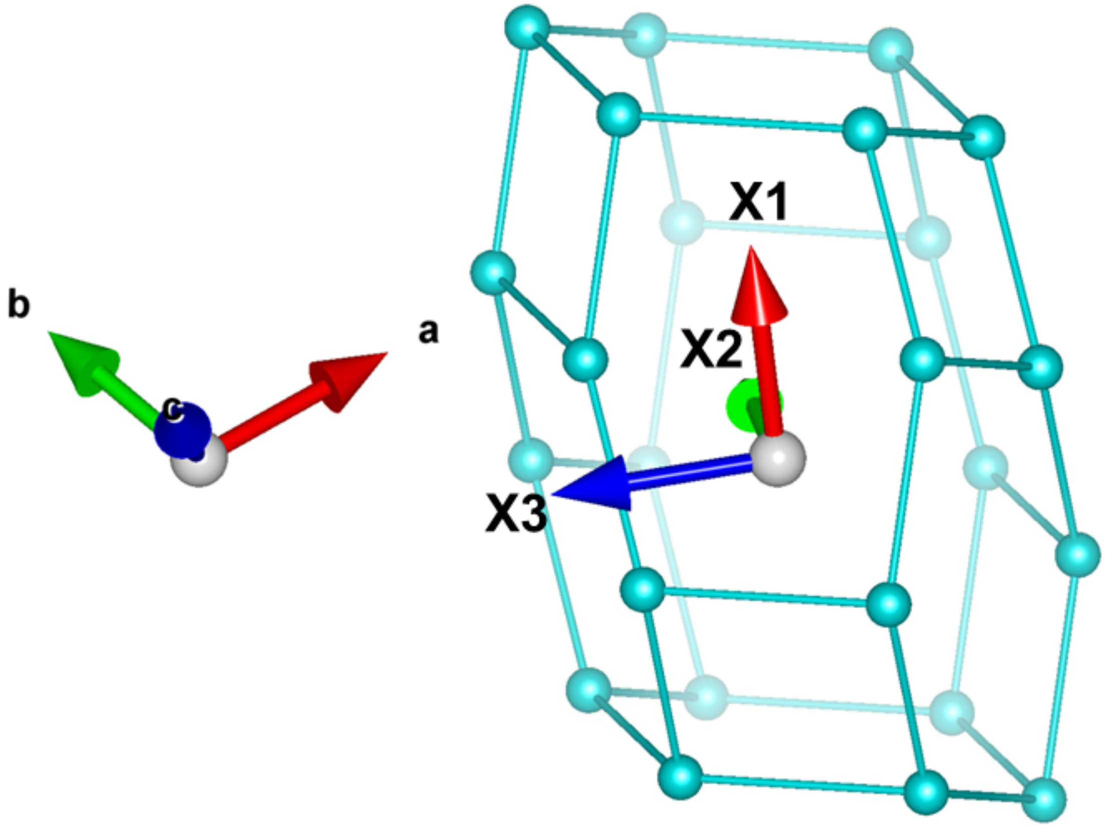

Figure S14. Representation of the sodalite-like cage of the ***np*** phase of ZIF-7 and the orientation of the orthogonal principal axes relative to the crystallographic axes.

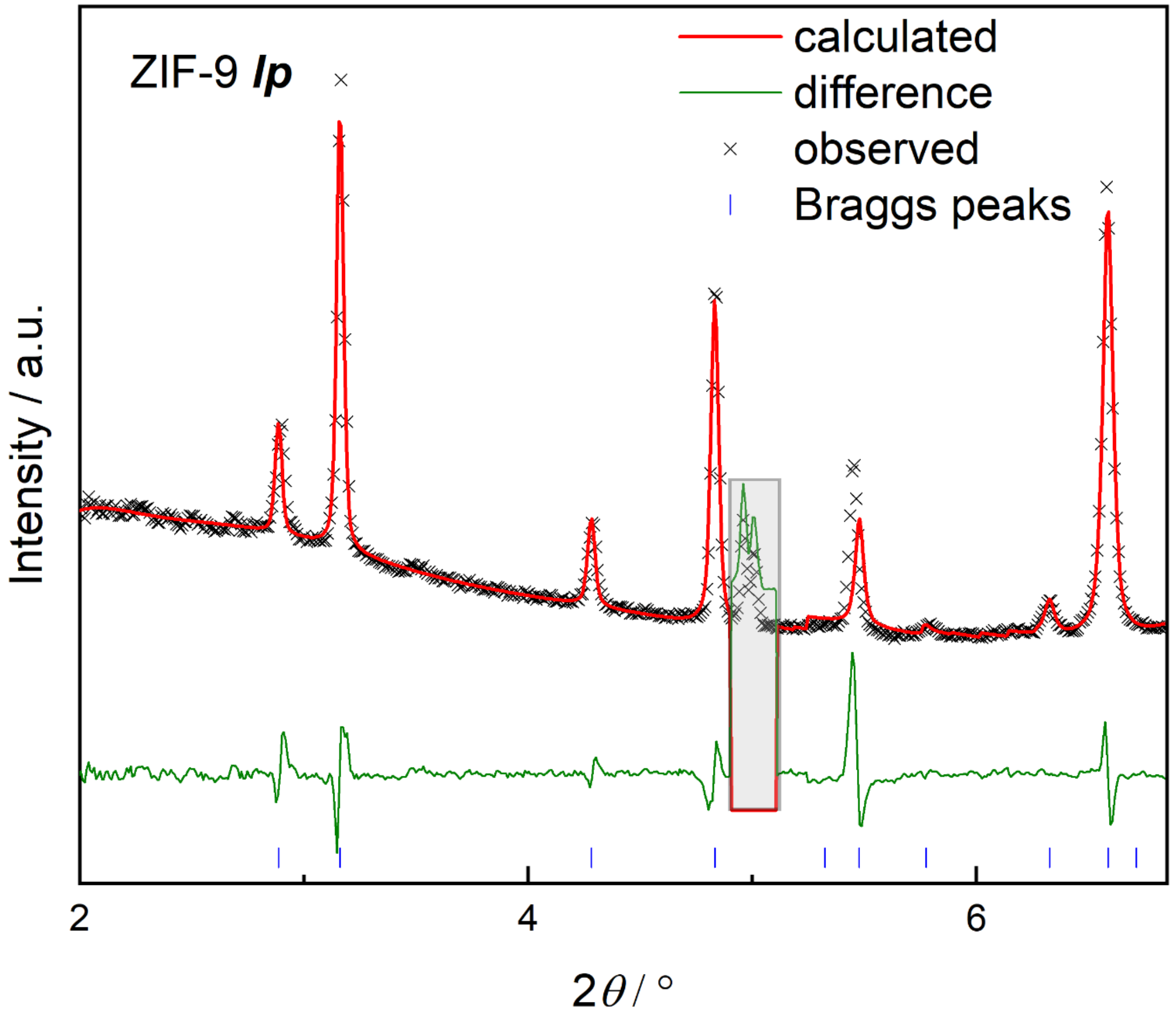

Figure S15. PXRD pattern with profile fit (Pawley method) of ZIF-9 ***lp*** phase obtained upon heating at 494 °C during VT-PXRD experiment. The data exceeding a 2θ value of 6.87° was excluded due to the presence of reflections attributed to the graphite dome of the hot stage (see Figure S14). The data ranged from 2θ 4.91° to 5.10° were also excluded as the reflections highlighted in grey stem from a contamination of the VT setup at high temperature measurements.

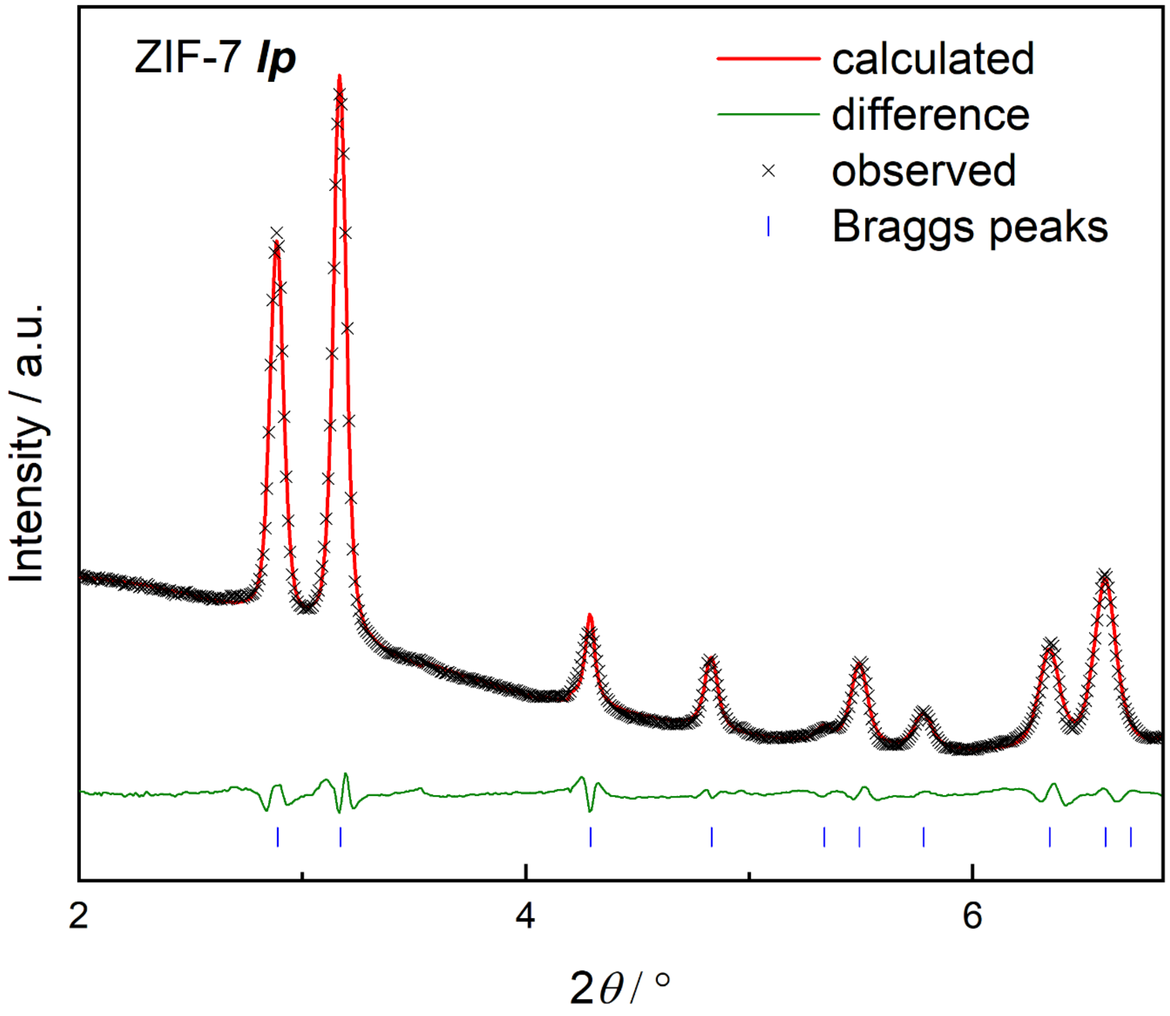

Figure S16. PXRD pattern with profile fit (Pawley method) of ZIF-7 ***lp*** phase obtained upon heating at 494 °C during VT-PXRD experiment. The data exceeding a 2θ value of 6.87 ° were excluded due to the presence of reflections attributed to the graphite dome of the hot stage (see Figure S16).

Table S12. Crystallographic data for the ***lp*** phases of ZIF-9 and ZIF-7 (space group $R\bar{3}$) determined via profile refinement of the VT-PXRD data collected at the temperatures of 494 °C and 450 °C, respectively.

Phase	*a* / Å	*b* / Å	*c* / Å	α / °	β / °	γ / °	*V* / Å^3	R_{wp}	R_{exp}	χ
**ZIF-9 *lp***	22.468(11)	22.468(11)	15.879(4)	90	90	120	6942(7)	5.35	148.44	0.04
**ZIF-7 *lp***	22.407(7)	22.407(7)	15.894(7)	90	90	120	6911(5)	1.42	147.26	0.01

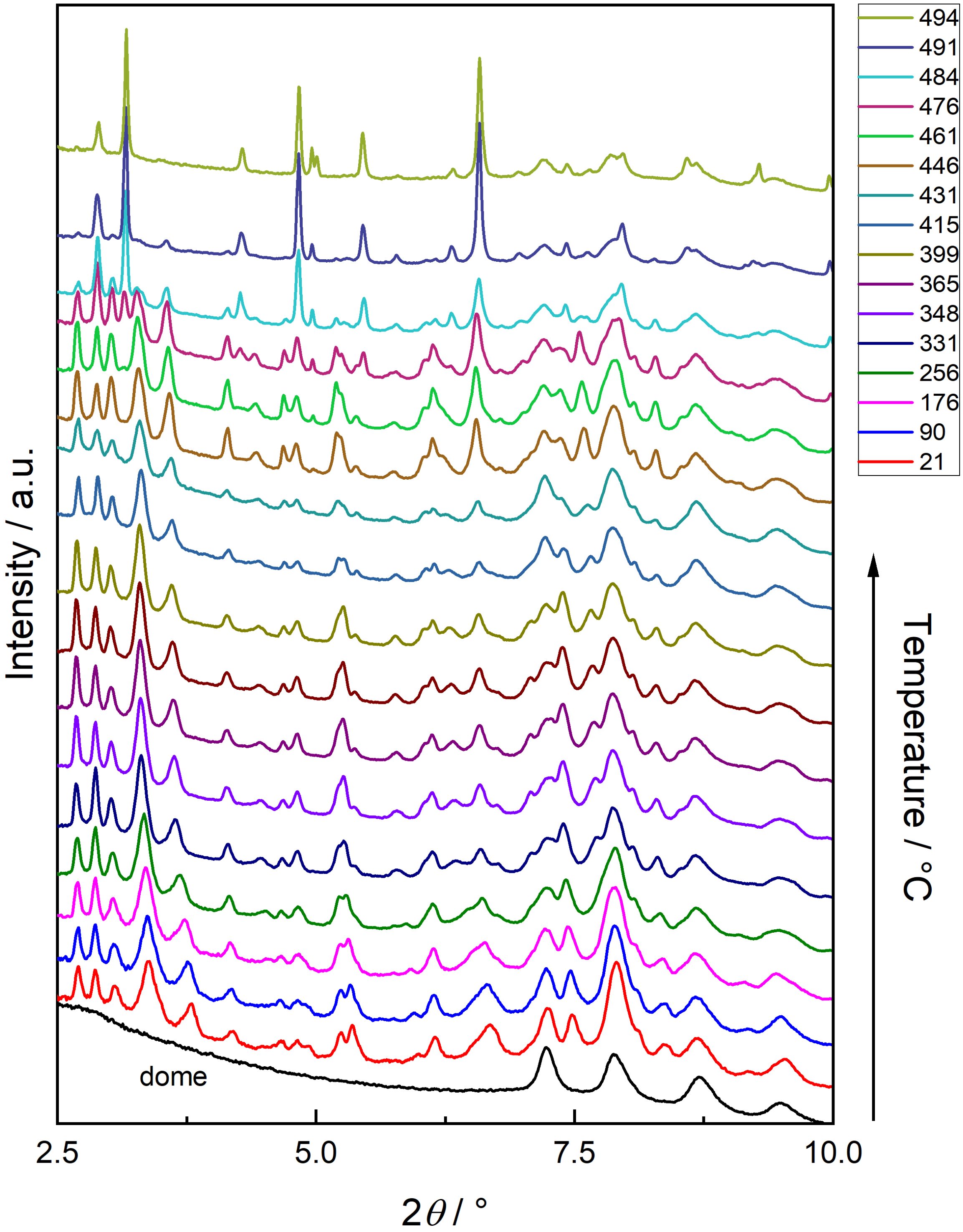

Figure S17. Stacked VT-PXRD patterns of ZIF-9 collected under heating from 21 °C (black) to 494 °C. (blue). For the sake of comparison, PXRD pattern of the dome has been included at the bottom (black).

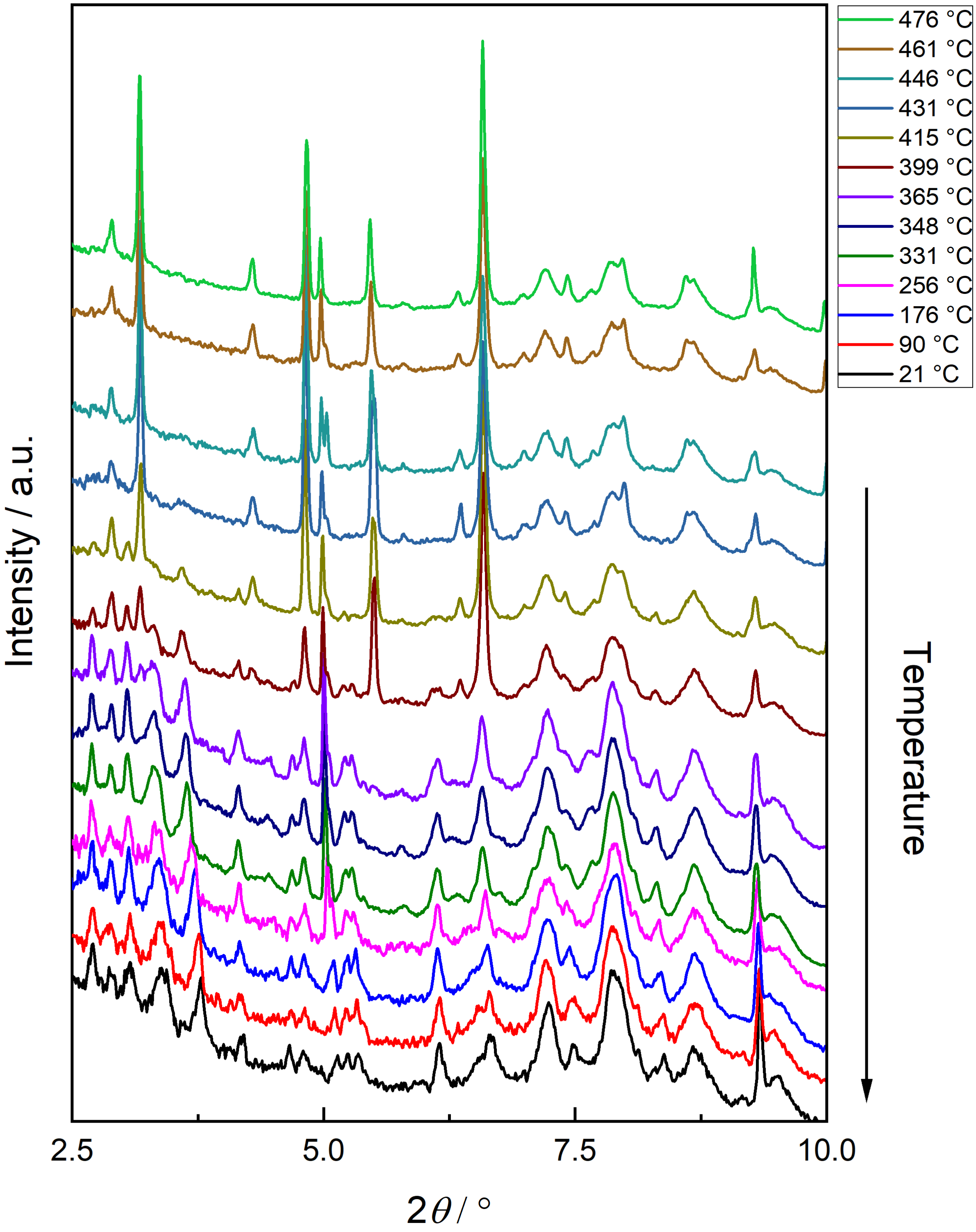

Figure S18. Stacked VT-PXRD patterns of ZIF-9 collected under cooling from 475 °C (black) to 21 °C (blue).

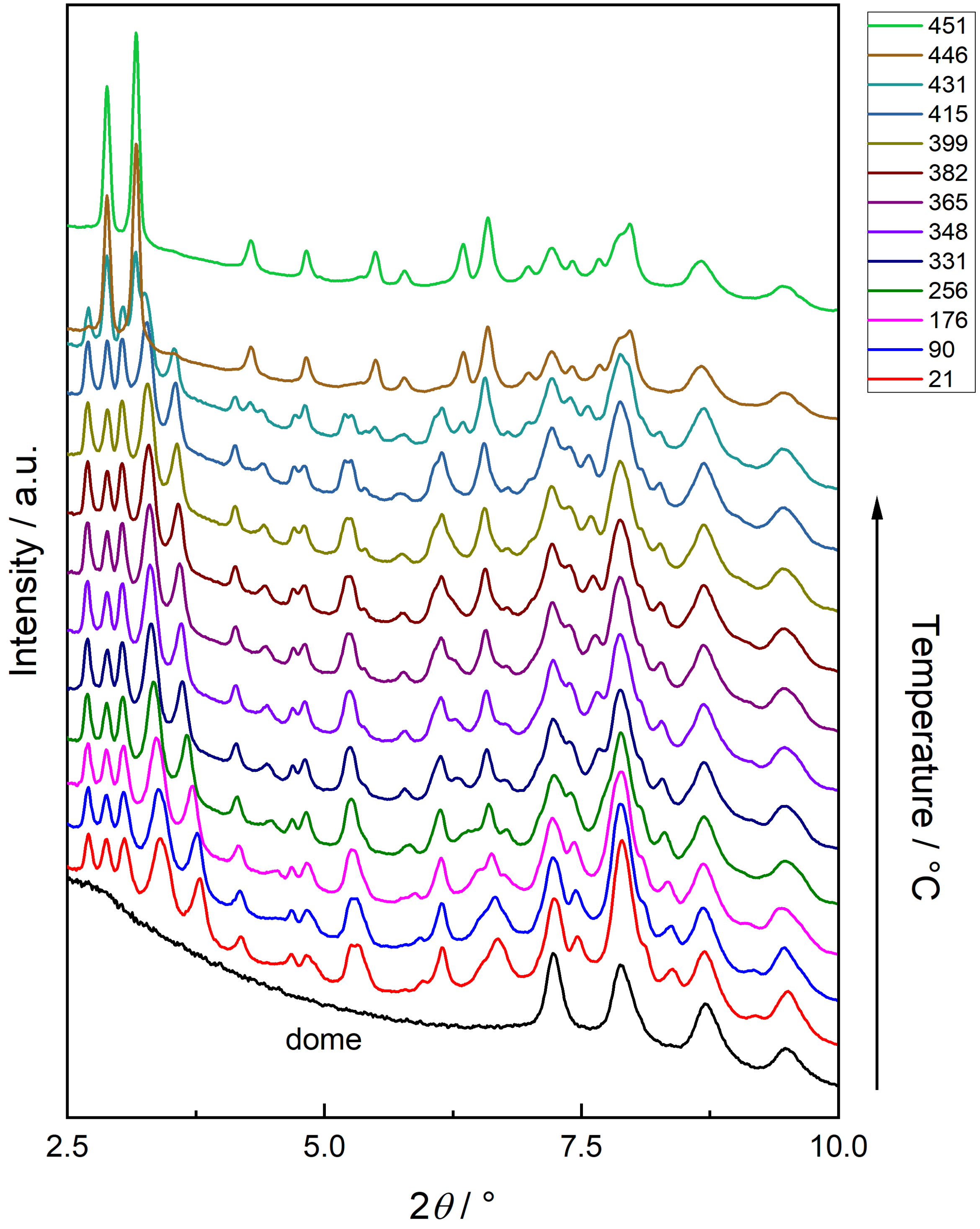

Figure S19. Stacked VT-PXRD patterns of ZIF-7 collected under heating from 21 °C (black) to 450 °C (brown). For the sake of comparison, PXRD pattern of the dome has been included at the bottom (black).

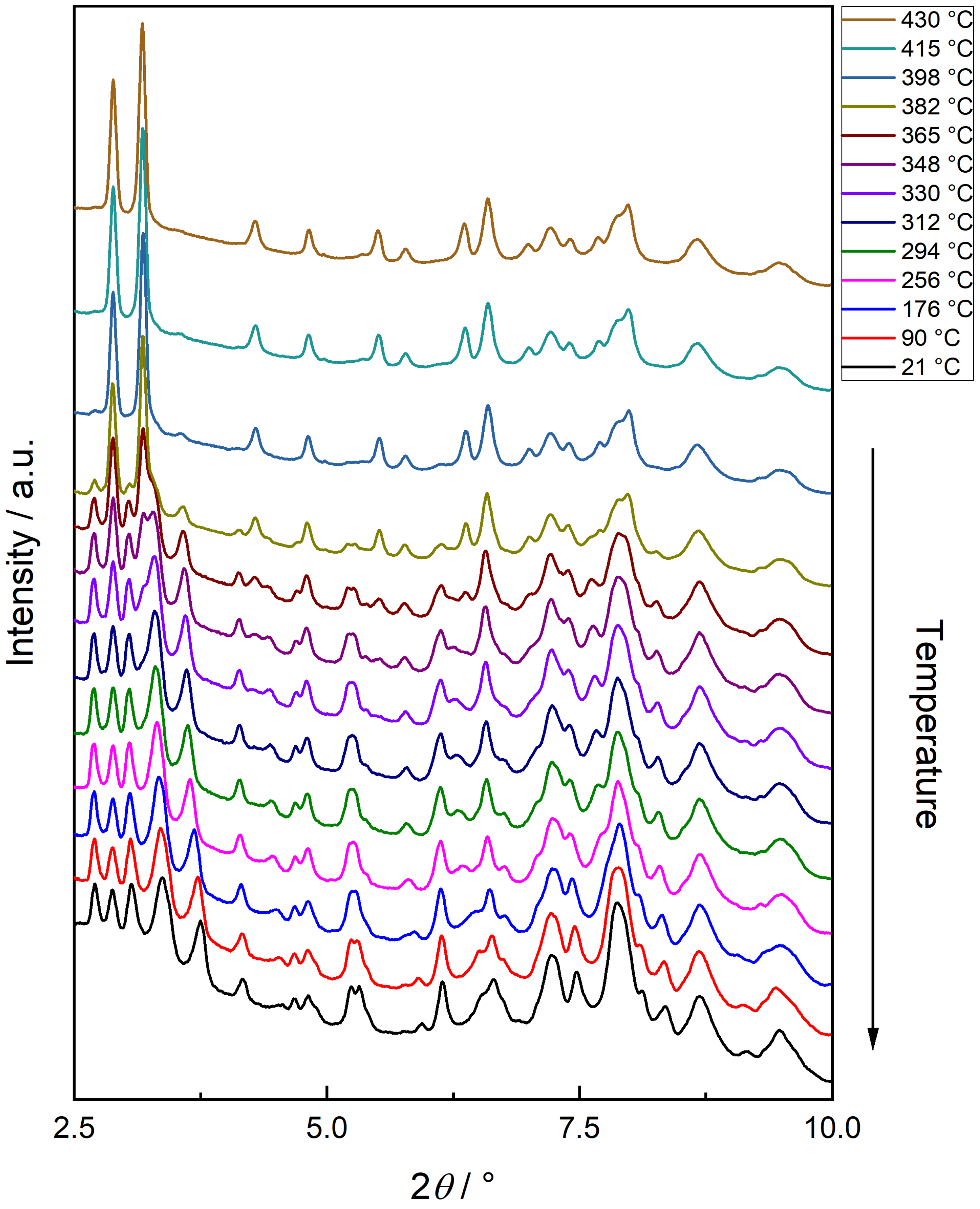

Figure S20. Stacked VT-PXRD patterns of ZIF-7 collected under cooling from 430 °C (brown) to 21 °C (black).

S4 References

1 M. O. Cichocka, J. Ångström, B. Wang, X. Zou and S. Smeets, *J. Appl. Crystallogr.*, 2018, **51**, 1652–1661.
2 W. Kabsch, *Acta Crystallogr. D Biol. Crystallogr.*, 2010, **66**, 125–132.
3 G. M. Sheldrick, *Acta Crystallogr. Sect. Found. Adv.*, 2015, **71**, 3–8.
4 W. Wan, J. Sun, J. Su, S. Hovmöller and X. Zou, *J. Appl. Crystallogr.*, 2013, **46**, 1863–1873.
5 E. Svensson Grape, *Zonedo.18029105*, 2025, 10.5281/zenodo.18029105.
6 G. M. Sheldrick, *Acta Crystallogr. A*, 2008, **64**, 112–122.
7 L.-M. Peng, *Micron*, 1999, **30**, 625–648.
8 G. S. Pawley, *J. Appl. Crystallogr.*, 1981, **14**, 357–361.
9 A. A. Coelho, *J. Appl. Crystallogr.*, 2018, **51**, 210–218.
10 Y. Okada and Y. Tokumaru, *J. Appl. Phys.*, 1984, **56**, 314–320.
11 P. W. Stephens, *J. Appl. Crystallogr.*, 1999, **32**, 281–289.
12 T. D. Kühne, M. Iannuzzi, M. Del Ben, V. V. Rybkin, P. Seewald, F. Stein, T. Laino, R. Z. Khaliullin, O. Schütt, F. Schiffmann, D. Golze, J. Wilhelm, S. Chulkov, M. H. Bani-Hashemian, V. Weber, U. Borštnik, M. Taillefumier, A. S. Jakobovits, A. Lazzaro, H. Pabst, T. Müller, R. Schade, M. Guidon, S. Andermatt, N. Holmberg, G. K. Schenter, A. Hehn, A. Bussy, F. Belleflamme, G. Tabacchi, A. Glöß, M. Lass, I. Bethune, C. J. Mundy, C. Plessl, M. Watkins, J. VandeVondele, M. Krack and J. Hutter, *J. Chem. Phys.*, 2020, **152**, 194103.
13 J. P. Perdew, K. Burke and M. Ernzerhof, *Phys. Rev. Lett.*, 1996, **77**, 3865–3868.
14 S. Grimme, J. Antony, S. Ehrlich and H. Krieg, *J. Chem. Phys.*, 2010, **132**, 154104.
15 S. Goedecker, M. Teter and J. Hutter, *Phys. Rev. B*, 1996, **54**, 1703–1710.
16 C. Hartwigsen, S. Goedecker and J. Hutter, *Phys. Rev. B*, 1998, **58**, 3641–3662.
17 M. Krack, *Theor. Chem. Acc.*, 2005, **114**, 145–152.
18 V. Haigis, Y. Belkhodja, F.-X. Coudert, R. Vuilleumier and A. Boutin, *J. Chem. Phys.*, 2014, **141**, 064703.
19 P. Gabbott, in *Principles and Applications of Thermal Analysis*, John Wiley & Sons, Ltd, 2008, pp. 1–50.
20 R. D. McGillicuddy, S. Thapa, M. B. Wenny, M. I. Gonzalez and J. A. Mason, *J. Am. Chem. Soc.*, 2020, **142**, 19170–19180.
21 M. He, J. Yao, L. Li, K. Wang, F. Chen and H. Wang, *ChemPlusChem*, 2013, **78**, 1222–1225.
22 K. S. Park, Z. Ni, A. P. Côté, J. Y. Choi, R. Huang, F. J. Uribe-Romo, H. K. Chae, M. O'Keeffe and O. M. Yaghi, *Proc. Natl. Acad. Sci.*, 2006, **103**, 10186–10191.
23 L. T. L. Nguyen, K. K. A. Le, H. X. Truong and N. T. S. Phan, *Catal. Sci. Technol.*, 2012, **2**, 521–528.
24 Z. Öztürk, J. P. Hofmann, M. Lutz, M. Mazaj, N. Z. Logar and B. M. Weckhuysen, *Eur. J. Inorg. Chem.*, 2015, **2015**, 1625–1630.
25 R. A. Klein, S. Shulda, P. A. Parilla, P. L. Magueres, R. K. Richardson, W. Morris, C. M. Brown and C. M. McGuirk, *Chem. Sci.*, 2021, **12**, 15620–15631.
26 P. R. Spackman, M. J. Turner, J. J. McKinnon, S. K. Wolff, D. J. Grimwood, D. Jayatilaka and M. A. Spackman, *J. Appl. Crystallogr.*, 2021, **54**, 1006–1011.
27 M. J. Cliffe and A. L. Goodwin, *J. Appl. Crystallogr.*, 2012, **45**, 1321–1329.
